# Long-term cultures of human pancreatic islets in self-assembling peptides hydrogels

**DOI:** 10.3389/fbioe.2023.1105157

**Published:** 2023-02-23

**Authors:** Amanda Marchini, Maria Gessica Ciulla, Barbara Antonioli, Alessandro Agnoli, Umberto Bovio, Virginia Visnoviz, Federico Bertuzzi, Fabrizio Gelain

**Affiliations:** ^1^ Institute for Stem-Cell Biology, Regenerative Medicine and Innovative Therapies (ISBReMIT), IRCCS Casa Sollievo della Sofferenza, San Giovanni Rotondo, Italy; ^2^ Center for Nanomedicine and Tissue Engineering (CNTE), ASST Grande Ospedale Metropolitano Niguarda, Milan, Italy; ^3^ Tissue Bank and Tissue Therapy Unit, ASST Grande Ospedale Metropolitano Niguarda, Milan, Italy; ^4^ Department of Biotechnology and Bioscience, University of Milan-Bicocca, Milan, Italy; ^5^ Department of Molecular Medicine, University of Pavia, Pavia, Italy; ^6^ Department of Diabetology, ASST Grande Ospedale Metropolitano Niguarda, Milan, Italy

**Keywords:** human pancreatic islets, self-assembling peptides, biomimetic nanomaterial, three-dimensional cell cultures, type 1 diabetes mellitus

## Abstract

Human pancreatic islets transplantation is an experimental therapeutic treatment for Type I Diabetes. Limited islets lifespan in culture remains the main drawback, due to the absence of native extracellular matrix as mechanical support after their enzymatic and mechanical isolation procedure. Extending the limited islets lifespan by creating a long-term *in vitro* culture remains a challenge. In this study, three biomimetic self-assembling peptides were proposed as potential candidates to recreate *in vitro* a pancreatic extracellular matrix, with the aim to mechanically and biologically support human pancreatic islets, by creating a three-dimensional culture system. The embedded human islets were analyzed for morphology and functionality in long-term cultures (14-and 28-days), by evaluating β-cells content, endocrine component, and extracellular matrix constituents. The three-dimensional support provided by HYDROSAP scaffold, and cultured into MIAMI medium, displayed a preserved islets functionality, a maintained rounded islets morphology and an invariable islets diameter up to 4 weeks, with results analogues to freshly-isolated islets. *In vivo* efficacy studies of the *in vitro* 3D cell culture system are ongoing; however, preliminary data suggest that human pancreatic islets pre-cultured for 2 weeks in HYDROSAP hydrogels and transplanted under subrenal capsule may restore normoglycemia in diabetic mice. Therefore, engineered self-assembling peptide scaffolds may provide a useful platform for long-term maintenance and preservation of functional human pancreatic islets *in vitro*.

## 1 Introduction

Type I Diabetes mellitus is a severe autoimmune disease caused by disruption of insulin-producing pancreatic β-cells. Loss of β-cells alters metabolic glucose homeostasis, leading to high blood glucose levels and chronic hyperglycemia (Katsarou et al., 2017). Among currently available therapies and since the seminal publication of the Edmonton protocol (Shapiro et al., 2000), intraportal transplantation of human pancreatic islets (hPIs) from multiorgan donors remains a consolidated and efficacious procedure for the replacement of β-cells. Before transplantation, hPIs are isolated from donor healthy pancreas thanks to a combination of mechanical and chemical methods that involve collagenase digestion. Isolated and purified hPIs are then transplanted into the liver via portal vein. This procedure has been successfully performed on patients with type I Diabetes, providing exogenous insulin independence for several years. Liver is considered the preferred transplantation site because the procedure is minimally invasive, with ease access and with a low rate of bleeding and thrombosis (Cayabyab et al., 2021). The outcome after 3-year follow-up reported a proper glucose control with no need of exogenous insulin administration, confirming an optimal glycemic homeostasis in patients (Bertuzzi et al., 2018b). At 5-year follow-up, islet transplantation led to insulin independence in almost all patients, without progress of secondary diabetic complications and with a stable kidney functionality (Bachul et al., 2021). However, a key component in successfully islets graft survival is a rapid revascularization that provides oxygen and nutritional supply to engrafted hPIs. Despite liver remains the preferred transplantation site, recent studies revealed that absence of extensive vascularization limits the in viability of transplanted hPIs in the long-term. Researches are investigating alternative transplantation sites (as intramuscular, omentum and subcutaneous sites) (Pellicciaro et al., 2017; Bertuzzi et al., 2018a; Yu et al., 2020), or strategies to promote rapid revascularization via angiogenic biomaterials (Vlahos et al., 2017; Fernandez et al., 2022).

Thus, some limitations hindered the success of transplantation, including global shortage of suitable donor organs, poor amount of purified islets after isolation procedure for clinics, time-limited islet functionality, lack of vascularization for oxygen and nutritional supply and morphological integrity due to basement membrane and extracellular matrix (ECM) disruption during enzymatic and mechanical digestion (Brandhorst et al., 2022). Basement membrane, and in general ECM, plays a crucial structural role, mediating adhesion and intracellular chemical signaling pathways, as well as β-cells survival and insulin secretion. Consequentially, islets undergo apoptosis, and quickly lose their integrity, morphology, and functionality due to the absence of ECM support and signaling (Cross et al., 2017; Townsend and Gannon, 2019). To maximize the success of transplantation in clinics, hPIs must be transplanted within 48–72 h of isolation (Ricordi et al., 2016; Cayabyab et al., 2021). Others focused on hPIs banking from multiple donors, thus developing protocols for pancreatic islets cryopreservation (Zhan et al., 2022).

However, the quest for strategies enabling long-term culturing of hPIs (e.g., pancreatic islets seeding in ECM-like scaffolds) is still a major goal for researchers. Recently developed protocols have demonstrated an alternative source of β-cells, obtaining a large-scale production of functional islet-like clusters from human pluripotent stem cells (Parent et al., 2022). Moreover, in recent years, organoid technology has attracted lots of attention for its unprecedent potential to reproduce *in vitro* structural and functional aspects of *in vivo* tissue/organ and bridge the gap between cellular- and tissue/organ-level in biological models (Takebe and Wells, 2019). Human pancreatic organoids could have opportunity to produce functional β-cells *in vitro* after human pluripotent stem cells differentiation process and could acts as source of islets for transplantation (Jiang et al., 2022; Yin et al., 2022). A tissue engineering approach is required for culturing insulin-producing cells in a ECM-like scaffold in three-dimensional (3D) constructs with the purpose to restore cell-matrix attachment and to maintain islet viability and insulin secretion functionality for long-time (Kumar et al., 2018; Elizondo et al., 2020; Abadpour et al., 2022; Perugini et al., 2022). Islet encapsulation within bioscaffold may provide an immune-isolation strategy for islet transplantation and engraftment, avoiding immune-suppression procedure to prevent rejection. Biomaterials can be also engineered to accommodate other type of cells (e.g., endothelial cells) with the aim to create a vascularized microdevice, exhibiting pro-angiogenic properties and improving transplantation outcome (Vlahos et al., 2017). Natural, synthetic or hybrid biomaterials with different methods of fabrication are currently used to incorporate insulin-secreting cells (Salg et al., 2019). Ideally, a scaffold should be a 3D porous biocompatible ECM-like matrix with a controlled degradation rate (Perez-Basterrechea et al., 2018). Among synthetic biomaterials, self-assembling peptides (SAPs) can be considered as suitable substrates for long-term islets maintenance for their tunable biomimetic and biomechanical properties in tissue engineering applications (Chen and Zou, 2019; Gelain et al., 2021). SAPs are small molecules, made of amino acids, capable of spontaneously self-assemble into various nanostructures, as nanofibers, nanotubes or nanovesicles, upon exposure to shifts of pH, temperature and osmolarity (Matson and Stupp, 2012; Pugliese and Gelain, 2017). Multiple functional bioactive motifs can be conjoined to SAPs to achieve the desired biomimetic properties and enhance cell-matrix interactions (Pugliese et al., 2018b).

Moreover, the introduction of branched SAP sequences ameliorated the mechanical stiffness, with the chance to mimic different living tissues (Pugliese et al., 2018a). SAPs used in this work have previously demonstrated an excellent cell attachment and differentiation potential in neural stem cell cultures (Gelain et al., 2012; Caprini et al., 2013; Marchini et al., 2020), a remarkable neuroregenerative potential *in vivo* (Marchini et al., 2019), and promising biocompatibility (Raspa et al., 2016); for all these reasons, SAPs could be considered promising scaffolds for other cell cultures and/or the regeneration of other tissues. In this regard, it has been reported that insulin-producing cells and neuronal cells share many characteristics, suggesting that these 2 cell types could also share the same growth and differentiation factors, due to common precursor during development process (Alpert et al., 1988). These similarities suggest that SAPs may have protective and beneficial effects on pancreatic islets cultures (Yuan et al., 2008). In this work, we investigated three different SAPs, namely HYDROSAP, FAQ and CK_1_, to generate 3D matrices for long-term maintenance (14-and 28-days) of hPIs. Freshly-isolated islets in suspension were considered positive control, while islets in suspension cultured for 14 and 28 days were considered as negative controls. In addition, we tested two types of culture media: MIAMI medium, a CMRL-based medium commonly used to maintain the pancreatic islets for few days before transplantation (Bertuzzi et al., 2018b), and medium enriched with growth factors (namely GF medium), that is a medium normally used for neural stem cell cultures (Gelati et al., 2013), containing epidermal growth factor (EGF) and basic fibroblast growth factor (bFGF). Embedded and free-floating islets were assessed for their morphology, viability, endocrine cellular content, and ECM molecules composition. Optimal ECM-like matrix was reached by HYDROSAP scaffold cultured in MIAMI medium, yielding results that demonstrated better islets preservation for 14 days, and then for 28 days: this was the case for cellular population composition (β-cells producing insulin, endocrine cells, and proliferative cells), apoptotic cells, endothelial cells, and ECM components (laminin, collagen I and collagen IV), as well as preservation of islets morphological features overtime. Finally, densely 3D-seeded hPIs in HYDROSAP scaffold were characterized to simulate the protocol developed for subsequent *in vivo* transplantation. Lastly, a preliminary *in vivo* study was conducted on nude diabetic mice, and islets maintained in culture for 14 and 28 days inside HYDROSAP were transplanted under kidney capsule.

## 2 Materials and methods

### 2.1 Isolation of human pancreatic islets

hPIs used in this study were isolated from six cadaveric multiorgan donors, in particular three women and three men, donor age 52.6 ± 3.6 years, BMI 25.5 ± 2.6 kg/m^2^, islets purity 82.5 ± 5.2%, according to the procedure previously described (Ricordi et al., 1988; Petrelli et al., 2011). The overall protocol has been approved by the Niguarda Cà Granda Ethics Board. Islets were isolated using the automated method previously described (Ricordi et al., 1988). Pancreata were obtained from multiorgan cadaveric donors utilizing cold perfusion. Exclusion and inclusion criteria were applied based on the Italian Guidelines. Briefly, pancreata were digested by a cold enzymatic blend solution of collagenase and thermolysin (Liberase MTF GMP Grade kit, Roche Diagnostics, Mannheim, Germany) reconstituted in Hank’s Balanced Salt Solution (HBSS, Euroclone, Italy) with 25 mM of HEPES. Subsequently, islets were purified with discontinuous polysucrose solutions at decreasing density 1.132, 1.108, 1.096, 1.060 and 1.037 g/L (Mediatech-Cellgro, VA, United States). Islets were counted by dithizone staining islet equivalent (IEQ) method (see “Dithizone staining” section) and they were cultured at 24°C, 20% O_2_, 5% CO_2_ in a humidified atmosphere in MIAMI Medium #1A (Mediatech-Cellgro, VA, USA) supplemented with Ciprofloxacin (Fresenius Kabi, Verona, Italy), or in serum-free medium in the presence of basic fibroblast growth factor (bFGF, PeproTech) and epidermal growth factor (EGF, PeproTech) at final concentrations of 10 ng/ml and 20 ng/ml, respectively.

### 2.2 Peptide synthesis and purification

All reagents and solvents used for the peptide synthesis were purchased in highest quality commercially available and used without further purification. All peptides were synthesized with solid-phase Fmoc-based chemistry on Rink amide resin (0.19–0.56 mmol/g, 100–200 mesh) using a Liberty Blue System synthesizer (CEM Corp, Matthews, NC, Canada). Peptides were cleaved from resin by addition of a freshly prepared mixture containing 92.5% TFA, 2.5% H_2_O, 2.5% DODt, 2.5% TIS. All synthesis were carried out on a 0.25 mmol in presence of a 0.2 M amino acid solution (in DMF), 1 M DIC (in DMF), and 1 M Oxyma (in DMF). The deprotection of Fmoc groups was determined by a 10% v/v of piperazine in 9:1 NMP/EtOH. The N-terminal acetylation (for CK_1_ and pureHYDROSAP components) was performed using 20% v/v solution of Ac_2_O (in DMF). The crude products were purified via reversed-phase chromatography by semi-preparative Waters binary HPLC (>96%) using a c18 RestekTM column and then lyophilized (Labconco, Kansas City, MO, USA). Purified peptides powder was subsequently dissolved in 0.1 M HCl to remove the presence of possible TFA salts. Three different SAPs were used for this study: pureHYDROSAP (Marchini et al., 2019; Marchini et al., 2020), FAQ (NH_2_-FAQRVPP-GGG-LDLKLDLKLDLK-CONH_2_) (Gelain et al., 2012) and CK_1_ (Ac-CGGLKLKLKLKLKLKGGC-CONH_2_) (Pugliese et al., 2018c; Ciulla et al., 2022). As previously described, pureHYDROSAP is composed by linear SAPs Ac-(LDLK)_3_-CONH_2_, Ac-KLPGWSGGGG-(LDLK)_3_-CONH_2_ (Caprini et al., 2013) and Ac-SSLSVNDGGG-(LDLK)_3_-CONH_2_ (Gelain et al., 2012) and branched SAP tris(LDLK)_3_-CONH_2_ (Pugliese et al., 2018a). For the experiments, pureHYDROSAP (abbrev. HYDROSAP), FAQ and CK_1_ powders were dissolved respectively to a final concentration of 2% (w/v), 5% (w/v) and 5% (w/v) in distilled water (Gibco).

### 2.3 Rheological studies

The rheological experiments were carried out on a rotational AR-2000 ex rheometer (TA Instruments, Waters Corp, Milford, CT, United States) equipped with an acrylic cone-plate geometry (diameter, 20 mm; angle, 1; truncation gap, 34 µm). All the tests were performed at 37°C using a Peltier plate in the lower plate of the instrument to assess a continuum control of the temperature during each test. All samples were dissolved in distilled water at the corresponding concentration and incubated overnight at 4°C. Thus, 584 mM sucrose solution were added to each peptide solution (ratio 1:1); then, MIAMI medium (instead of the volume of hPIs) were included to obtain a final concentration of 0.76% (w/v), 1.9% (w/v) and 1.9% (w/v) for HYDROSAP, FAQ and CK_1,_ respectively. Each sample (50 µL) was gently placed on the middle of the Peltier plate. A lid on the top serves to protect sample from the water evaporation. To evaluate the storage (G′) and loss (G″) moduli increments, frequency-sweep experiments were recorded as a function of angular frequency (0.1–100 Hz) at a fixed strain of 1% after a 3 h time-sweep experiment (performed at 1 Hz constant angular frequency) in presence of D-PBS (1X). Stress–strain sweeps were performed in the range 0.01%–1,000% of strain. Thixotropy of peptides was performed with shear-thinning tests by a series of peak holds at constant shear rates. All data were performed in triplicate and the results were processed with OriginPro 2019 (OriginLab Corporation, Northampton, MA, United States). Graphs were reported in the Supplementary Figure S1).

### 2.4 Culture of human pancreatic islets inside nanostructured scaffold

Cell cultures were prepared within 24/48 h after receiving islets. hPIs were incapsulated inside hydrogels or plated in suspension in 48-well with two different type of culture media: MIAMI medium #1A (Mediatech-Cellgro, VA, United States) (a CMRL-based culture medium commonly used in clinical trial for islet culture before transplantation) or GF medium (a serum-free medium with bFGF and EGF growth factors). 25 IEQ (low density) or 500 IEQ (high density) were incapsulated inside SAPs, previously dissolved in distilled water and diluted with 584 mM sucrose solution (ratio 1:1). A droplet of 25 µL was placed onto glass coverslip in 48-well, medium was added to start SAP gelation and to obtain free-floating samples. Same concentration of islets was used for samples in suspension in 48-well. In these conditions, the samples were maintained in culture up to 14-days (T14) and 28-days (T28) at 24°C, 20% O_2_, 5% CO_2_ in a humidified atmosphere. As positive control, suspensions of islets in MIAMI medium or GF medium were maintained in culture for 1 day (T1). hPIs incapsulated insides hydrogel were monitored individually during culture time and brightfield images from day 1 to day 28 were acquired weekly *via* Zeiss light microscope at ×5 magnification.

### 2.5 Dithizone staining

Dithizone (Sigma-Aldrich, St Louis, MO, United States) is a zinc chelating agent, well-known to selectively stain pancreatic islets in a brownish red color. During islets purification and after 1 (T1), 14 (T14) and 28 days (T28) post-isolation, an aliquot of suspension of hPIs was stained with dithizone to track the islet morphology during time and to observe the purity of preparation. 200 µL of Dithizone solution was added to 1 ml of islets suspension in culture media for 10 min at room temperature. Following incubation time, three washes in D-PBS 1X (Gibco) was performed and images were acquired using Zeiss light microscope at ×5 magnification.

### 2.6 Diameter analysis

Images acquired *via* Zeiss light microscope of hPIs encapsulated inside SAPs and hPIs in suspension after Dithizone staining were processed *via* NIH-ImageJ software and Axiovision software. Diameter of each islet at each timepoint were calculated and reported.

### 2.7 Immunofluorescence staining

hPIs in suspension and encapsulated in hydrogel maintained in culture for 1, 14 and 28 days were fixed in paraformaldehyde (PFA) 2% and 4% (w/v in PBS) and cryosectioned at 50 µm-thick *via* Cryostat (Histo-Line Laboratories). Sections were permeabilized with 0.3% Triton X-100 for 10 min at 4°C and blocked with 10% normal goat serum (NGS, Gibco) for 1 h at room temperature. The following primary antibodies were used: rabbit anti-insulin (1:300, ThermoFisher), mouse anti-chromogranin (1:100, ThermoFisher), mouse anti-glucagon (1:8000, Sigma-Aldrich), rabbit anti-Ki67 (1:750. Novus Biologicals), rabbit anti-vWF (1:500, DakoCytomation), mouse anti-fibroblast (1:200, Acris Antibodies), rabbit anti-collagen IV (1:100, Cedarlane), mouse anti-collagen I (1:2000, Sigma-Aldrich), rabbit anti-laminin (1:30, Sigma-Aldrich). To reveal primary antibodies, the following secondary antibodies were used: goat anti-rabbit Cy3 (1:1,000, Jackson), goat anti-mouse Cy3 (1:1,000, Jackson), goat anti-rabbit Alexa 488 (1:1,000, Invitrogen) and goat anti-mouse Alexa 488 (1:1,000, Invitrogen). Cell nuclei are stained with HOECHST 33342 (1:500, Molecular Probes).

### 2.8 Apoptosis

Tunel assay (*In situ* cell death detection kit fluorescein, Roche) was performed to detect and quantify apoptotic cells. The protocol was performed following the manufacturer’s instructions. Briefly, slices were permeabilized with 0.3% Triton X-100 for 10 min at 4°C and incubated with Tunel reaction mixture (1:10 enzyme in label solution) for 1 h at 37°C. Cell nuclei are stained with HOECHST 33342 (1:500, Molecular Probes).

### 2.9 Images acquisition and data analysis

Whole hPIs were imaged in fluorescence at × 20 and × 40 magnification *via* Zeiss Microscope connected to Apotome System and image analysis was performed with Fiji-ImageJ NIH-software. A minimum of three independent experiments were performed for each experimental condition, timepoint and marker. For insulin, chromogranin, glucagon, Ki67 and Tunel Assay markers, quantitative analysis were performed by counting manually positive cells for each marker, compared to total cells contained in a single islet and stained with Hoechst. For vWF, Fibroblast, Laminin and Collagen I and IV, fluorescent images were converted into binary images and reactivity area were quantified by measuring the number of positive pixels for each marker, compared to the total area of hPIs.

### 2.10 Experimental design for pilot *in vivo* experiment

All the animal procedures and ethical revision were performed according to the current Italian Legislation (Legislative Decree 4 March 2014, n.26) enforcing the 2010/63/UE Directive on protection of animals used for biomedical research. *In vivo* protocol was approved by Institutional Animal Care and Use Committee (IACUC number 63/2022-PR). 18 CD-1 Nude Male Mice, weight 27/30 g (approx. 7 weeks) were divided into three groups: 1) six animals received freshly-isolated hPIs (positive control); 2) six animals received hPIs pre-cultured in SAPs for 2 weeks; 3) six animals received hPIs pre-cultured in SAPs for 4 weeks hPIs used for *in vivo* experimentations came from two different donors. After diabetes induction, 1,500 IEQ hPIs were transplanted under kidney capsule of diabetic mice, by inspiring a well-known surgical technique (Szot et al., 2007). Mice were monitored for 50 days post-surgery by studying non-fasting blood glucose level (non-fasting BGL) and body weight every other day, and intraperitoneal glucose tolerance test (IPGTT) at two- and 4-week post-transplantation. Normoglycemia was achieved when blood glucose levels scored values < 200 mg/dl. At the end of *in vivo* experiment, mice were sacrificed by cervical dislocation and engrafted kidneys were excised, fixed, and cryo-sectioned to visualized with immunohistochemical studies the presence of engrafted hPIs *via* human insulin markers. See Supplementary Figure S1 and Methods for more details.

### 2.11 Statistical analysis

Results are reported as means ± SD in all graphs. Data were processed using GraphPad Prism seven software for *in vitro* and *in vivo* experiments. *In vitro* tests were performed in triplicate: three different islets preparations, derived from three different donors, are used for each condition (time-points, type of medium and scaffold) for each marker. Statistical analyses were evaluated by Two-way ANOVA followed by Tukey’s multiple comparison. Comparison between low density and high density of hPIs were performed by two-way ANOVA followed by Bonferroni’s multiple comparison test. Statistical significance was set at *p*-value < 0.05.

## 3 Results and discussion

### 3.1 Fabrication of bioscaffolds for *in vitro* 3D hPIs cultures

Biomaterials are key structural elements in 3D cell cultures to tune cell attachment, growth, differentiation and function (Marchini and Gelain, 2022). The ECM composition and organization to be mimicked by the biomaterials can differ and depends on the tissue of origin. Pancreatic ECM, enriched in laminin, collagen IV and VI, and fibronectin, provides structural and biochemical support to β-cells (Townsend and Gannon, 2019). Islet-ECM interaction regulates survival, insulin secretion, β-cells proliferation, differentiation, and preservation of round islet morphology (Stendahl et al., 2009). Current islet isolation techniques disrupt the native islet ECM, leading to faster cell death and dysfunction. Biomaterials, and in particular SAPs, can act as efficient synthetic substitute**s**, since they can be tailored according to specific *in vitro* and/or *in vivo* applications by changing mechanical, physicochemical and biological parameters (Marchini and Gelain, 2022). SAPs used in this work were widely applied in the field of neural stem cells (Gelain et al., 2012; Pugliese et al., 2018c; Marchini et al., 2019; Marchini et al., 2020). It is well-known that brain tissue is very soft, with a stiffness ranging from 0.1kPa to 1 kPa (Ciulla et al., 2022): pancreas stiffness lays in the similar range (Guimarães et al., 2020), with healthy pancreas tissue ∼900 Pa and with increased ECM stiffness in pancreatic cancer (∼2,900 Pa) (LeSavage et al., 2022). Three different SAPs (HYDROSAP, FAQ and CK_1_) were evaluated in terms of rheological analysis to investigate their viscoelastic and mechanical properties, as well as to compare their characteristics to that of pancreatic tissue. In order to mimic viscoelastic properties of normal human pancreatic tissue, thixotropy (Supplementary Figure S1, on the left), and stiffness, in terms of storage modulus (G′) and loss modulus (G″) (Supplementary Figure S1, on the right) were evaluated. The rheological characterization revealed that storage moduli in HYDROSAP, FAQ and CK_1_ enclose G’ values ranging to 826 ± 75 Pa for FAQ, 1,583 ± 165 for HYDROSAP, and 5,440 ± 421 Pa (CK_1_), showing a range of stiffness close to the ECM surrounding of human pancreatic cells. In other words, the SAPs scaffolds used in this study were satisfactorily similar (in term of rigidity) to the healthy pancreas tissue (HYDROSAP, FAQ), or to tumorigenic pancreas (CK_1_). Lastly, thixotropy experiments described the capacity of these materials to recover their original behavior after reversible breaking and recovering of self-assembled network.

**FIGURE 1:**
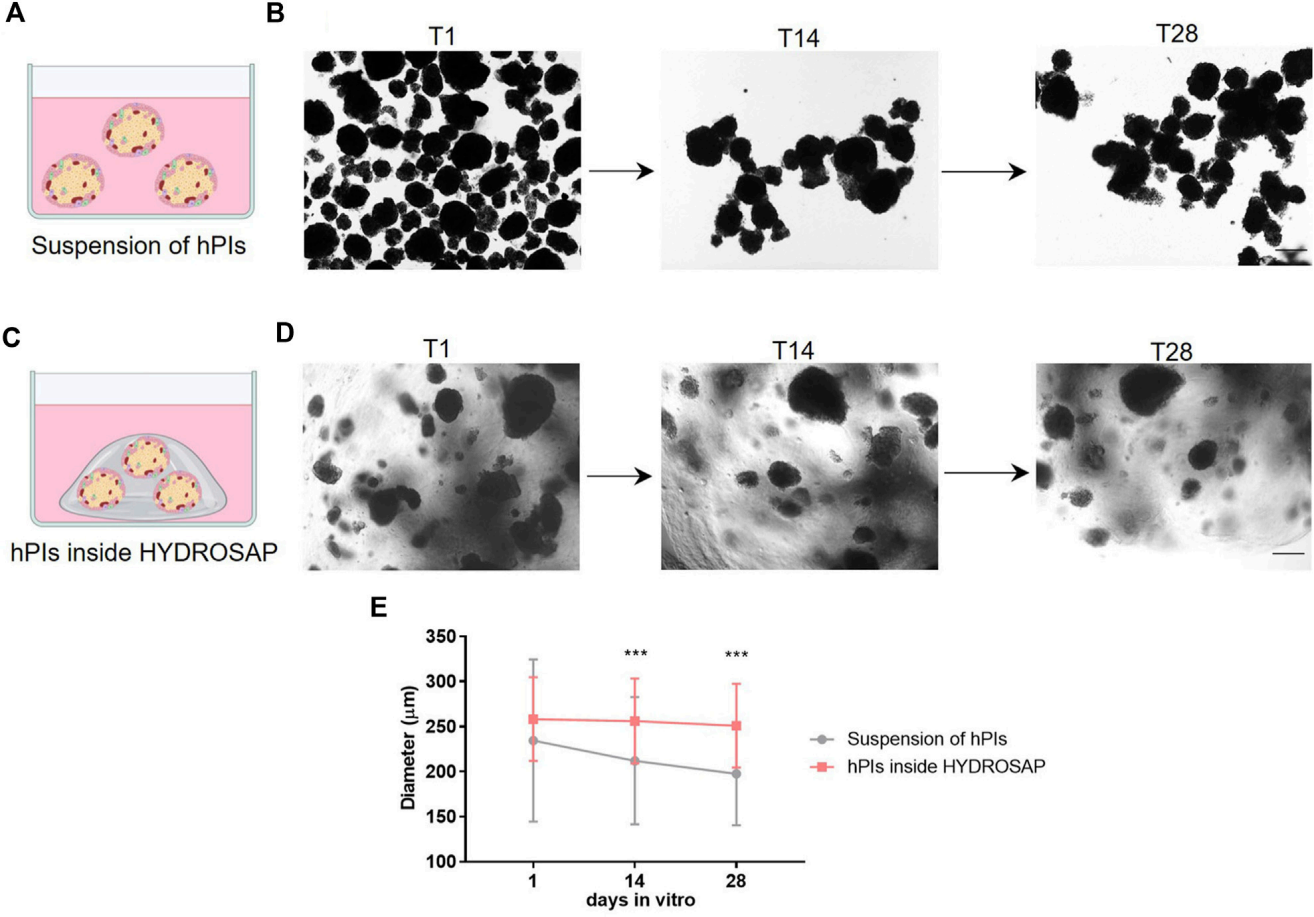
*In vitro* experimental design and hPIs morphological evaluation. **(A)** In standard condition, hPIs were maintained in free-floating suspension in culture medium. **(B)** Time-course of hPIs integrity captured in brightfield microscopy: round hPIs cluster at 1-day post-isolation (T1) turned into hPIs clustered together losing their original morphology and integrity after 14 (T14) and 28 (T28) days. **(C)** hPIs embedded in SAP scaffolds mimicking structural features of extracellular matrix. **(D)** Morphology and integrity of embedded hPIs inside HYDROSAP was monitored through the weeks via inverted microscope, showing no morphological alteration over weeks. **(E)** Islets diameter quantification over time till 28-days post-isolation for free-floating hPIs and hPIs seeded in HYDROSAP hydrogels. Graph shows mean ± SD of triplicate samples per each time point and statistical difference between groups (two-way ANOVA followed by Tukey post-hoc test; ***p < 0.001). Scale bar, 100 µm.

In *vitro* studies, standard hPIs cultures were maintained in free-floating condition as a control group (Figure 1A). In Figure 1B, hPIs morphology and integrity are monitored and captured in brightfield microscopy. At 1-day post-isolation (T1), hPIs appeared as distinct round clusters; after 14 (T14) and 28 (T28) days, hPIs clustered together, by losing their original morphology and integrity. At T28, the edge appeared completely jagged and islets loss their morphological integrity, due to ECM disruption during enzymatic isolation. Dithizone staining, routinely applied in free-floating condition for assessing islet quality, purity, and morphology, demonstrated a robust decrease of β-cells functionality (Khiatah et al., 2019), underlined by a steady switch of islets color from reddish to brown one (Supplementary Figure S2). To overcome these problems, functionalized SAPs were used as building blocks to mimic biofunctional and structural features of ECM (Marin and Marchesan, 2022) and support hPIs, by controlling numerous cellular processes, such as cell adhesion (Figure 1C). In this regard, islets morphology was monitored through the weeks via an inverted microscope. As an example, in Figure 1D are shown hPIs embedded in HYDROSAP, but similar observations also were found for FAQ and CK_1_ (Supplementary Figure S2). As depicted in Figure 1D, hPIs were not subjected to morphological alteration during weeks. SAPs prevented cluster formation and hPIs edge remained clearly defined. To corroborate the above-mentioned qualitative data, the alteration in hPIs diameter was monitored overtime up to 28-days post-isolation (Figure 1E). As expected, hPIs embedded inside HYDROSAP kept stable their dimension, starting from 258.29 ± 46.36 μm at T1, to 256.17 ± 47.03 μm at T14, and 250.95 ± 46.42 μm at T28. Conversely, hPIs in suspension, in absence of a structural support, completely lost their initial morphology and their diameter decreased significantly from 234.49 ± 89.94 μm at T1, to 212.08 ± 70.51 μm at T14, and 197.5 ± 57.11 μm at T28. Indeed, at 14 and 28 DIV (days *in vitro*) statistical analysis showed significant differences in islets diameter between suspension of hPIs and hPIs inside HYDROSAP (***p < 0.001). Diameter conservation was also assessed for islets embedded inside FAQ and CK_1_ scaffold, as depicted in Supplementary Figure S3 and Supplementary Table S1.

### 3.2 Long-term *3D* cultures of hPIs (14-days)

**FIGURE 2:**
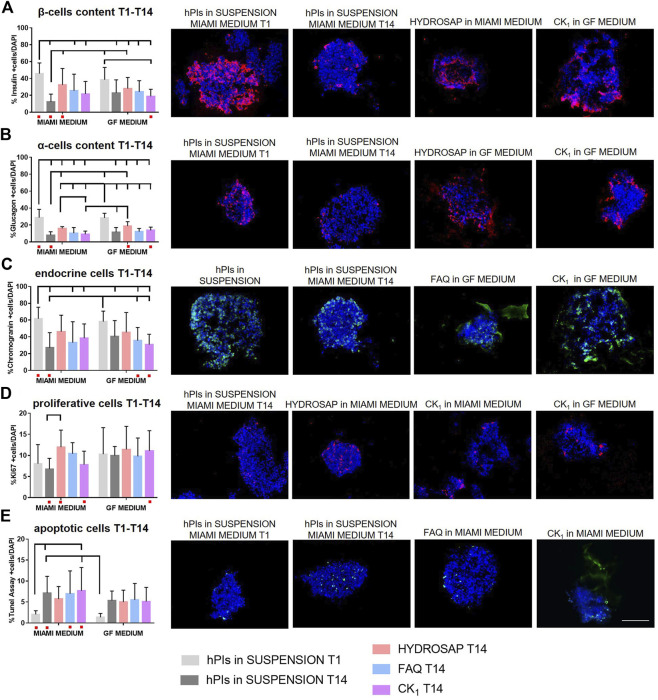
Cellular components characterization of hPIs cultured for 14-days in HYDROSAP, FAQ and CK_1_ scaffolds, or plated in suspension in MIAMI medium or GF medium. Freshly-isolated hPIs in suspension cultured in MIAMI medium for 1 day (T1) were considered as positive control; conversely, hPIs maintained in suspension for 14-days in MIAMI or GF media were chosen as negative controls. Immunofluorescence analysis and related representative images for **(A)** β-cells producing insulin in red, **(B)** α-cells producing glucagon in red, **(C)** endocrine component in green, **(D)** cells in proliferation in red, and **(E)** apoptotic cells in green show the preservation for 14-days of β-cells content, endocrine cells, and proliferative component and a controlled percentage of apoptotic cells in scaffold-embedded hPIs, especially for HYDROSAP. No significant difference was highlighted between two culture media. Cell nuclei are stained in blue. Images illustrate islets in selected culture conditions, depicted in each graph with a red dot. Data are presented as mean ± SD, two-way ANOVA with Tukey post-hoc test: statistical differences were evaluated among all groups and specified in Supplementary Table S2 (*n* = 3; *p < 0.05; **p < 0.01; ***p < 0.001). Scale bar, 100 µm.

As demonstrated in the previous section, HYDROSAP, FAQ and CK_1_ could act as ECM-like scaffolds, due to their mechanical properties similar to pancreatic tissue, and their ability to maintain overtime morphological conformation of hPIs. First, cellular content in all samples at 14 DIV was analyzed with immunofluorescence stainings against the main islet hormones (insulin and glucagon), endocrine cells, proliferating cells and apoptotic cells (Figure 2). Islets were 3D seeded in HYDROSAP, FAQ and CK_1_ scaffolds, or plated in suspension, with two different cell culture media: MIAMI medium and GF medium. As a positive control, freshly-isolated suspensions of islets were cultured in MIAMI media for 1 day (T1). Islets maintained in suspension for 14-days in MIAMI or GF media (without mechanical support) were chosen as negative controls. Percentage of cells producing insulin (Figure 2A and Supplementary Table S2) decreased drastically (***p < 0.001) in suspension in MIAMI medium at T14, compared to freshly-isolated islets T1 (45.84 ± 12.59% and 12.18 ± 9.29%, respectively). Conversely, in free-floating condition, GF medium seems to maintain more efficiently insulin content for 14-days, thanks to bFGF and EGF growth factors that increases insulin secretion (Wang et al., 2001; Lee et al., 2008). The effect of 3D matrices was then investigated. Only HYDROSAP scaffold cultured in MIAMI medium was able to preserve percentage of insulin cells (32.35 ± 19.51%); indeed, HYDROSAP was the only hydrogel not showing significant differences with freshly-isolated islets at T1. Percentage of glucagon decreased in all conditions with ***p < 0.001 (Figure 2B; Supplementary Table S2) compared to freshly-isolated islets in MIAMI medium (28.76 ± 9.75%) and in GF medium (28.56 ± 5.34%). Glucagon component is not a crucial hormone for patients with Type 1 Diabetes: its decreased amount can be considered as less crucial to the success of islets transplantation. The percentage of chromogranin, a protein located in secretory vesicles of endocrine cells, was also performed (Figure 2C). Percentage of chromogranin-positive endocrine-cells is conserved in islets embedded in HYDROSAP in MIAMI medium with 46.22 ± 19.68% and in GF medium with 45.33 ± 23.64%, if compared with freshly isolated samples (61.73 ± 13.67% for MIAMI medium and 58.31 ± 12.40% for GF medium). Conversely, islets maintained in FAQ and CK_1_ obtained results comparable to islets in suspension for 14-days (27.04 ± 17.99%) (Supplementary Table S2). The proliferative capacity of all endocrine cells in islets was also tested (Figure 2D). Compared to T1 (8.06 ± 4.49%), proliferation rate was preserved overtime in all substrates, without significant differences between samples (Supplementary Table S2). Quantitative analysis of apoptotic cells inside islets grown into different substrates was achieved (Figure 2E). The rate of cell apoptosis is relatively higher in all conditions overtime, compared to hPIs suspension at T1 (2.07 ± 0.86%), with the highest percentage of apoptotic cells reached by islets maintained in CK_1_ in MIAMI medium for 14-days (7.96 ± 5.53%, **p < 0.01, Supplementary Table S2). Despite some detected apoptosis, the obtained results denoted a negligible degree of cytotoxicity.

**FIGURE 3:**
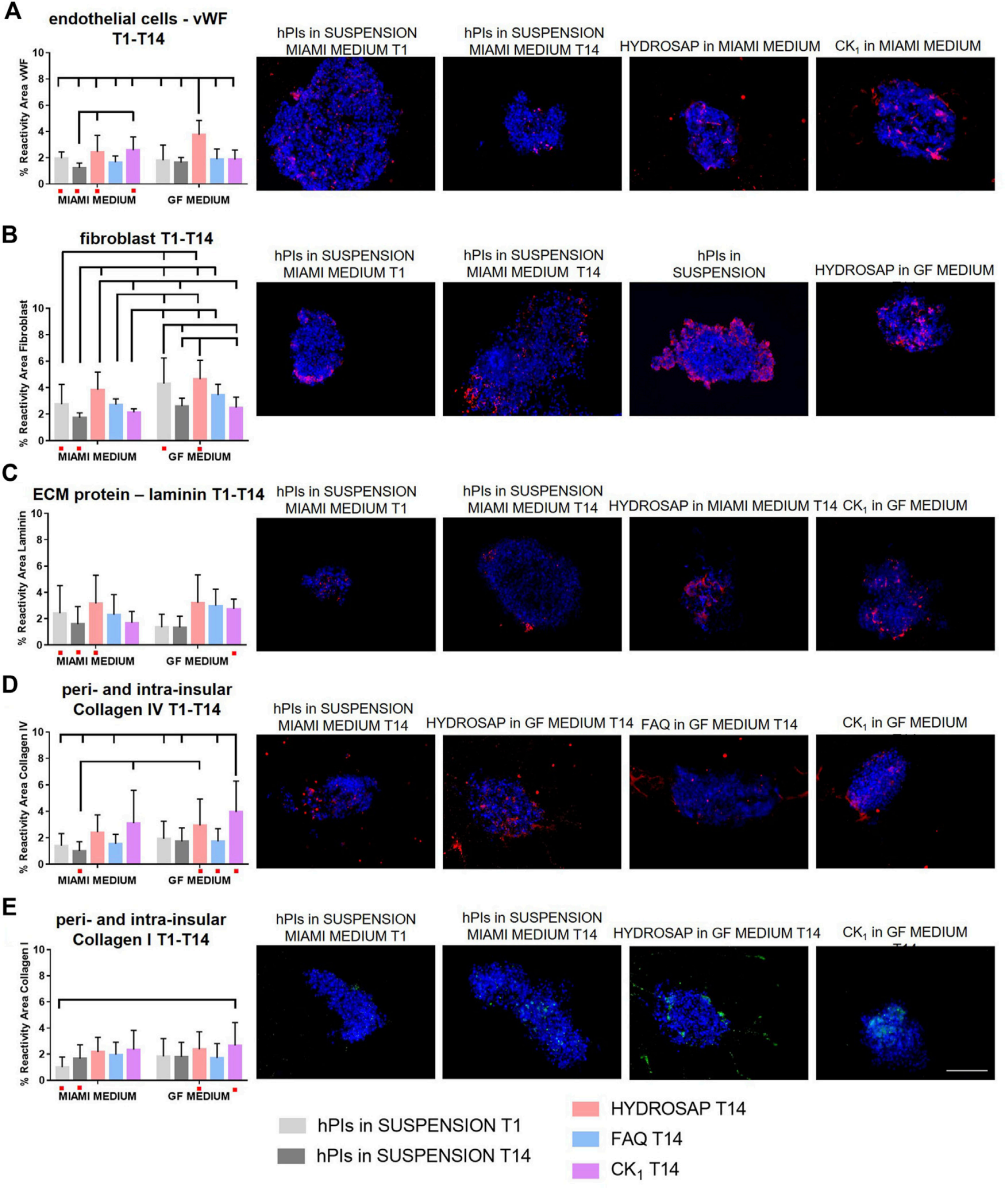
Characterization of main ECM components, fibroblast cells and endothelial cells in hPIs at 14 days *in vitro* in HYDROSAP, FAQ and CK_1_ scaffolds or in suspension in MIAMI or GF media. Fresh-isolated hPIs (T1) were considered as positive control, while hPIs in suspension for 14-days in MIAMI or GF media as negative controls. Graphs and related images show percentage of reactivity area for **(A)** vWF in red, **(B)** fibroblast in red, **(C)** laminin in red, **(D)** collagen IV in red, and **(E)** collagen I in green, showing a stable expression in HYDROSAP scaffold (with no significant differences between culture media) for all these markers. Cell nuclei are stained in blue. Images illustrate islets in selected culture conditions, depicted in each graph with a red dot. Data are presented as mean ± SD, two-way ANOVA with Tukey *post hoc* test: statistical differences were evaluated among all groups and specified in Supplementary Table S3 (*n* = 3; *p < 0.05; **p < 0.01; ***p < 0.001). Scale bar, 100 µm.

In order to characterize different ECM components inside hPIs and to evaluate their preservation right after their isolation (T1) and at 14 DIV, immunofluorescence analyses were performed for the main ECM components of pancreas (laminin, collagen IV, and collagen I), for fibroblast cells and for endothelial cells (Figure 3). Isolation procedure disrupts internal vascularization, innervation of islets, intra-islet ECM and peripheral islet ECM, by interrupting fundamental cell-matrix signaling (Stendahl et al., 2009). Biomimetic scaffolds could act as supporting elements by mimicking ECM and preserve, or even induce production, of some ECM components. Pancreatic islets are extensively vascularized and vascular network is essential for their function and survival (Xiong et al., 2020). Moreover, islets re-vascularization is a key elements for graft success following islet transplantation in the treatment of Type 1 Diabetes (Brissova and Powers, 2008). Von Willebrand factor (vWF) is a key component of blood and is produced by endothelial cells (Figure 3A). At 14 DIV, vWF expression did not decrease if compared to freshly-isolated islets: on the contrary a significantly higher expression in HYDROSAP with GF media (3.75 ± 1.08%) was found, probably due to the presence of EGF, that plays an important role in angiogenesis and significantly influence differentiation and proliferation of vascular endothelial cells (Chen et al., 2020). The combination of the multi-functionalized microenvironments of HYDROSAP and the exposure to EGF probably induced this considerable increase. Indeed, percentage of reactivity area in hPIs embedded into HYDROSAP in GF medium is almost doubled compared to freshly-isolated islets (1.96 ± 0.48%, ***p < 0.001); in hPIs cultured in HYDROSAP and CK_1_ in MIAMI medium (2.44 ± 1.25%, 2.58 ± 1.00%, respectively), vWF values were still higher than negative control (1.23 ± 0.36%) with **p < 0.01 and ***p < 0.001, respectively (Supplementary Table S3). Human islets are surrounded by a peri-islet capsule consisting of a layer of fibroblast and collagen fibers, produced by fibroblast. This capsule is closely associated with basement membrane which provides physical stability and anchorage to the islets (Banerjee, 2020). Fibroblasts are a cell population that participate on ECM and connective tissue formation, maintain tissue homeostasis, and preserve structural components. It was demonstrated that in a subcutaneous islet transplantation model, fibroblasts is associated with a faster graft re-vascularization, a higher insulin-positive area and a lower cell death (Perez-Basterrechea et al., 2017). Fibroblasts too are degraded during the enzymatic and mechanical isolation process of hPIs. As expected, bFGF, enclosed in GF media, enhanced fibroblast content in all hPIs encapsulated inside scaffolds (Figure 3B; Supplementary Table S3). The effect of bFGF was also observed in the short time in suspension of hPIs at T1 (4.30 ± 1.94%), compared to the counterpart in MIAMI medium (2.74 ± 1.50%, **p < 0.01). HYDROSAP scaffold showed significantly higher percentages of fibroblast positive cells (3.83 ± 1.34% in MIAMI medium, 4.63 ± 1.43% in GF medium). On the other hand, FAQ and CK_1_ preserved fibroblast component just like in positive control.

Together with collagens, laminin plays an important role among the ECM components of hPIs. If collagens provide structural stiffness to tissue, laminins maintain integrity and conformation of tissue structure. In human islets, laminins are involved in cytoskeletal remodeling, contractility, and control β-cells differentiation and insulin secretion (Llacua et al., 2018). Laminin resulted well-conserved in hPIs seeded in all SAPs (Figure 3C). Lasty, it was evaluated the most abundant ECM molecule in hPIs, i.e., collagens. Collagen improves endocrine functions, survival, and proliferation, and it is also a principal target during digestion for islet isolation (Riopel and Wang, 2014; Sakata et al., 2020). Collagen IV is a major component of the peri- and intra-islet ECM, modulates ECM stiffness, promotes cell survival, and decreases insulin production at high concentrations (Kaido et al., 2006). hPIs cultured for 14-days inside CK_1_ samples in GF medium (3.95 ± 2.33%) obtained an unexpected higher value in reactivity area compared to initial situation at T1 (1.41 ± 0.91%, ***p < 0.001) (Figure 3D and Supplementary Table S3). For the reason mentioned before, probably this result justified the lower values of β-cells in CK_1_-seeded islets (Figure 1A). The other SAPs preserved Collagen IV very well, without significant differences compared to hPIs in suspension at T1. Similar results were obtained for Collagen I (Figure 3E), that is normally localized within and around islets such as collagen IV, promotes islet cellular survival and differentiation and also improves β cell function (Wieland et al., 2021). CK_1_ scaffold supplemented with GF medium significantly enhanced Collagen I content (reactivity area) compared to freshly isolated islets (2.66 ± 1.76% and 1.01 ± 0.76%, respectively), while no decreased expression was observed for other SAPs (Supplementary Table S3). Some studies have demonstrated that abnormal collagen upregulation is associated with cancer cell proliferation. Such doubled production of Collagen I and IV in cells cultured in CK_1_ scaffold for 14-days could be due to the peculiar rigidity of CK_1_ that matches with tumorigenic pancreatic tissue. Additional analysis on collagen fibers orientation could be significant in predicting cancer (Angel and Zambrzycki, 2022). Tumor-ECM is more abundant, condensed, and stiffer than the ECM in the surrounding healthy tissue. Moreover, oriented collagen fibers around tumor cells, identification of specific collagen organization patterns and evaluation of collagen-associated biomarkers are indicators of tumor progression (Baldari et al., 2022; Song et al., 2022).

In summary, a stable expression of insulin, endocrine component, proliferative cells, apoptotic cells, vWF expression, fibroblast, laminin, collagen IV and I, between freshly-isolated islets (positive control) and islets embedded into HYDROSAP for 14-days was achieved. Moreover, if compared to islet in suspension for 14 days in MIAMI medium (negative control), a higher percentage of insulin, glucagon, proliferative cells, vWF, fibroblast and collagen IV and a lower percentage of apoptotic cells was observed. No relevant differences between the 2 cell culture media were detected, even if the GF medium looked like to increase the percentage of some ECM components. As previously described, FAQ and CK_1_ have not fulfilled all requirements. Still, CK_1_, with its high storage modulus (5,440 ± 421 Pa, Supplementary Figure S1), could be further investigated for application in the field of pancreatic tumors. Indeed, enhanced ECM stiffness and the upregulation of collagen production were demonstrated to be closely correlated to cancer progression (Zhang et al., 2021; Kpeglo et al., 2022). Alternatively, since changing SAPs concentration influence SAPs storage and loss moduli (Caprini et al., 2013; Gelain et al., 2021) it is very likely that a fine-tuning of CK concentration could allow to obtain a scaffold that better matches native pancreas biomechanics.

Encouraged by the obtained results, the culture time was extended till 28-days to evaluate the feasibility of long-term cultures of hPIs. Among the analyzed SAPs, HYDROSAP was chosen as the best substrate for 3D hPIs cultures and therefore additionally tested in this work.

### 3.3 Extended (28-days) hPIs *in vitro* cultures

**FIGURE 4:**
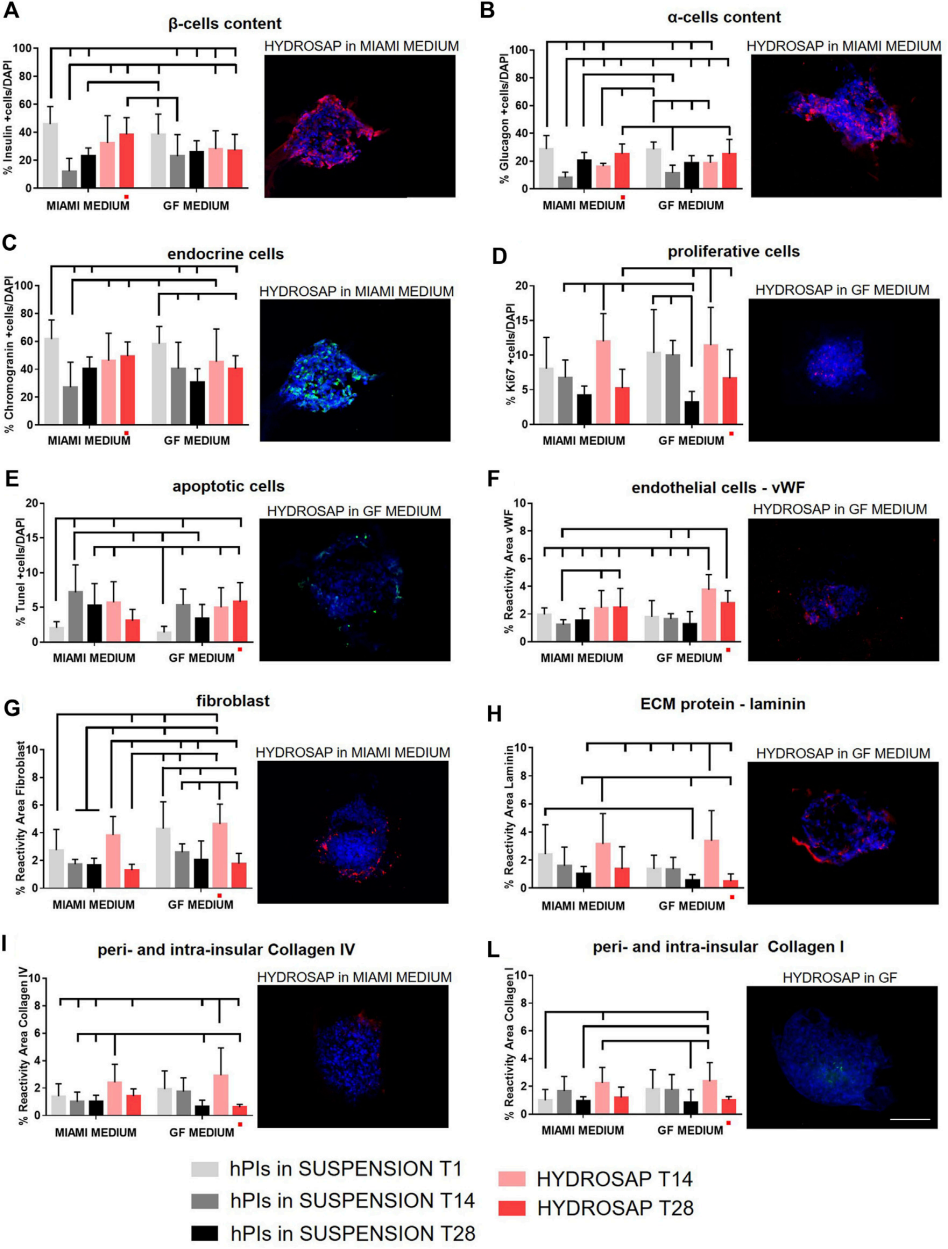
Long-term time-tracking of hPIs in suspension or encapsulated in HYDROSAP hydrogel. Islets were cultured for 1 day (T1), 14 days (T14) and 28 days (T28) and fed with MIAMI or GF media. Graphs and related images represent the percentage of positive cells for **(A)** insulin (red), **(B)** glucagon (red), **(C)** endocrine cells (green), **(D)** proliferative cells (red), displaying steady values (28-days) especially for hPIs cultured in MIAMI medium, with a stable percentage of apoptotic cells (**E**, in green) over time. In long-term cultures, also the percentage of reactivity area for **(F)** endothelial cells in red, **(G)** fibroblast in red, **(H)** laminin in red, **(I)** collagen IV in red and (**L**) collagen I in green were evaluated: vWF and collagen I remained stable in HYDROSAP with both MIAMI and GF media, while fibroblast, laminin, and collagen IV decreased drastically at 28-days in HYDROSAP with GF media, while it stabilized in HYDROSAP with MIAMI medium. Cell nuclei are stained in blue. Images illustrate islets in selected culture conditions, depicted in each graph with a red dot. Data are presented as mean ± SD, two-way ANOVA with Tukey post-hoc test: statistical differences were evaluated among all groups at different timepoints and specified in Supplementary Table S4 (*n* = 3; *p < 0.05; **p < 0.01; ***p < 0.001). Scale bar, 100 µm.

All marker analyses were repeated at 28 DIV (T28) and previous data at T1 and T14 were reported in graphs as reference (Figure 4). Only HYDROSAP scaffold was chosen to encapsulate hPIs for 28-days, while FAQ and CK_1_ were excluded from the study because of their sub-optimal performances. Figure 4 shows the overall *in vitro* characterization: endocrine markers (insulin, glucagon, and chromogranin), cells in proliferation, apoptotic cells and markers related to endothelial cells, fibroblast, and physiological ECM molecules (laminin, collagen I and IV). β-cells content (Figure 4A; Supplementary Table S4) in hPIs suspension in MIAMI medium at T1 scored 45.83 ± 12.59% of total cells; this amount decreased drastically in islets in suspension at T28 (23.25 ± 5.57%, ***p < 0.001), but it was better preserved in HYDROSAP-embedded hPIs in MIAMI medium (38.50 ± 12.01%), without significant differences with hPIs at T1. On the other hand, only 27.11 ± 11.41% is reached by hPIs in HYDROSAP in GF medium with a significant difference compared to positive control (***p < 0.001). Similarly, glucagon content is preserved over weeks *in vitro* compared to fresh-isolated islets (28.76 ± 9.75%) with values between 25.33 ± 7.00% for hPIs-embedded HYDROSAP in MIAMI medium and 25.18 ± 10.43% for hPIs-embedded HYDROSAP in GF medium at T28 (Figure 4B; Supplementary Table S4).

A similar trend observed for endocrine cells: the percentage of chromogranin-positive endocrine-cells was conserved in long-term cultures of HYDROSAP-embedded hPIs in MIAMI medium (49.33 ± 10.36%) (Figure 4C; Supplementary Table S4). In GF medium chromogranin reached a percentage of 40.47 ± 9.20%, significantly lower than in positive control (61.73 ± 13.67%, **p < 0.01). Cellular proliferative rate underwent a slow reduction in all groups: the percentage of 11.97 ± 4.01% (at T14 in HYDROSAP MIAMI) is halved at T28 in the same culture conditions (5.24 ± 2.73%, *p < 0.05); identically, hPIs cultured in HYDROSAP in GF medium decreased from 11.42 ± 5.47% at T14 to 6.66 ± 4.13% at T28 (Figure 4D; Supplementary Table S4).

On the other hand, the percentage of apoptotic cells (TUNEL assay) in all conditions remained stable (Figure 4E; Supplementary Table S4) if compared to results obtained at T14, suggesting a controlled apoptosis at 28 DIV (Figure 1E).

Immunofluorescence analysis of ECM components showed a vWF expression of HYDROSAP-embedded hPIs in MIAMI medium similar to freshly-isolated islets; conversely, it was found a significantly higher expression in HYDROSAP-embedded hPIs at T14 (3.75 ± 1.08%) and T28 (2.81 ± 0.86%) in GF medium if compared to hPIs in MIAMI medium at T1 (1.96 ± 0.48%) and T14 (1.23 ± 0.37%) and to hPIs in GF medium at T1 and T14 (1.80 ± 1.16% and 1.63 ± 0.38%, respectively) (Figure 4F and Supplementary Table S4). As mentioned before, this result is probably due to the combination of HYDROSAP scaffold and the presence of EGF in the GF culture medium (Figure 1F).

On the other hand, the high fibroblast content reported in HYDROSAP in GF media at T14 (4.63 ± 1.43%), decreased drastically after 28-days of culture (1.77 ± 0.75%, ***p < 0.001) (Figure 4G; Supplementary Table S4). Moreover, reactivity area for fibroblast marker is reduced also inside HYDROSAP maintained in MIAMI medium at T28 (1.31 ± 0.43%), compared to its T14 counterpart (3.83 ± 1.34%, ***p < 0.001).

Finally, extracellular matrix component (laminin, collagen IV, and collagen I) (Figure 4H–L; Supplementary Table S4) are well-conserved in HYDROSAP-embedded hPIs maintained in MIAMI medium for 28-days: statistical analysis did not show significant difference with freshly isolated hPIs and with same condition at T14. Conversely, the percentage of laminin (Figure 4H) and Collagen IV (Figure 4I) decreased drastically at 28 days in HYDROSAP with GF media (0.51 ± 0.52% and 0.61 ± 0.20% respectively) compared to the same conditions at T14 (3.36 ± 2.15% for laminin with **p < 0.01 and 2.93 ± 2.00% for Collagen IV with ***p < 0.001, Supplementary Table S4)

In conclusion, hPIs cultured *in vitro* have obtained excellent results in term of cellular components. When 3D cultured in HYDROSAP scaffold, all main cellular elements are preserved up to 28 DIV if compared to fresh-standard hPIs cultures. At 28-days vWF and collagen I remained stable inside HYDROSAP both in Miami medium and GF medium. The percentage of fibroblast, laminin, and collagen IV decreased drastically at 28-days in HYDROSAP with GF media and remained almost stable in HYDROSAP cultured in MIAMI medium. Accordingly, HYDROSAP in MIAMI medium significantly preserved functionality of α and β cells, controlled cell apoptosis, preserved ECM components, maintained a rounded hPIs morphology and islets diameter *in vitro* up to 4 weeks.

### 3.4 Long-term culture of densely seeded hPIs

**FIGURE 5:**
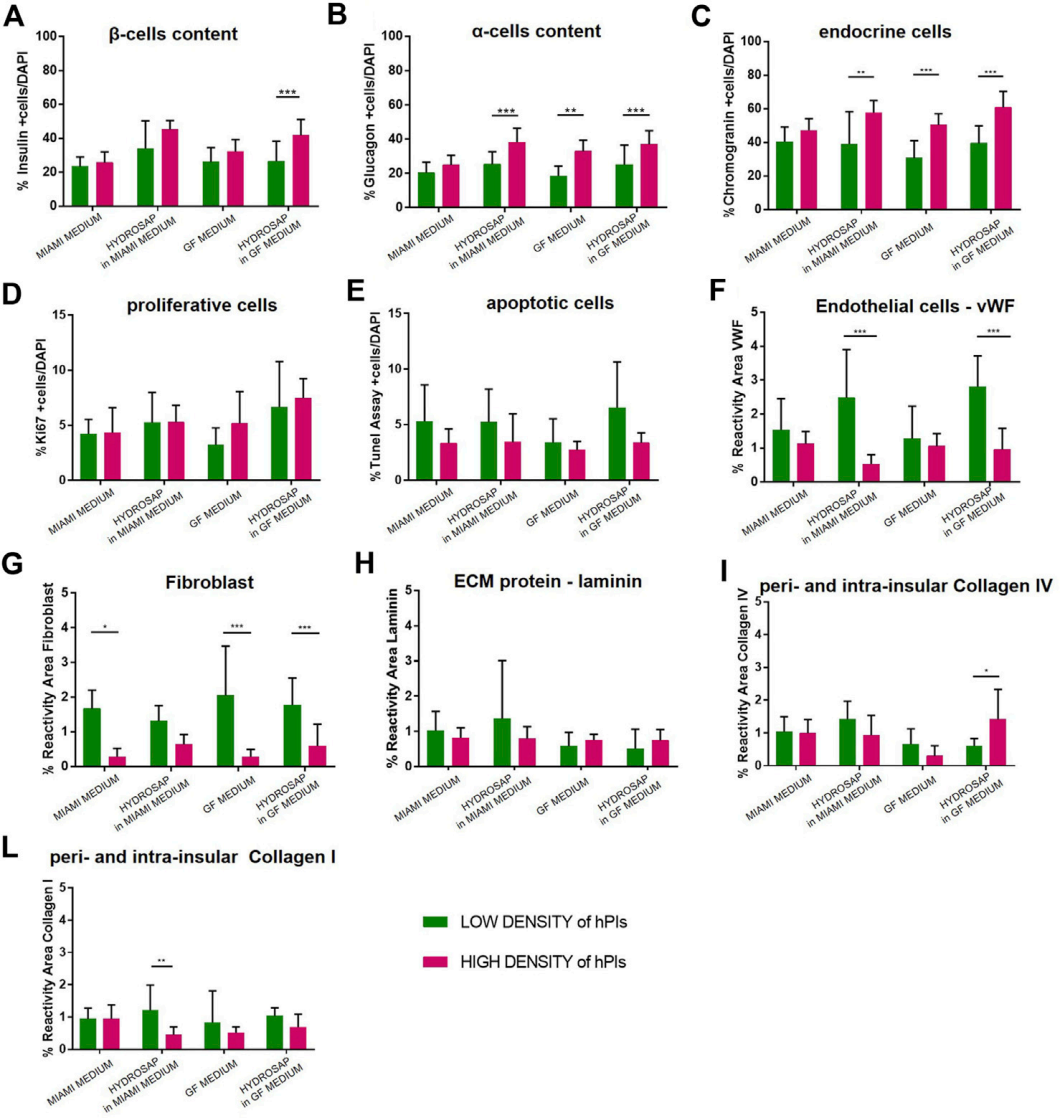
Comparison between Low-Density (25IEQ) and High-Density (500IEQ) cultures of hPIs in suspension in MIAMI medium, in suspension in GF medium, embedded in HYDROSAP with MIAMI medium and embedded in HYDROSAP with GF medium after 28-days of culture. hPIs in suspension in MIAMI and GF medium cultured for 28-days are considered as negative controls. Graph shows mean ± SEM of triplicate samples per conditions for percentage of **(A)** β-cells content, **(B)** α-cells content, **(C)** endocrine cells, **(D)** proliferative cells, **(E)** apoptotic cells, **(F)** endothelial cells, **(G)** fibroblast, **(H)** laminin, **(I)** collagen IV, and (**L**) collagen I, indicating an increased percentage of α-cells, β cells and endocrine fraction, followed by a lower concentration of ECM components. Experiments were performed in triplicate for each condition, and statistical analysis (two-way ANOVA with Bonferroni post-hoc test) showed significant differences between the two analyzed conditions (*p < 0.05; **p < 0.01; ***p < 0.001).

Abovementioned results were conducted with 25IEQ inside each 3D scaffold. Now, islets concentration was boosted to 500 IEQ to evaluate islets viability and functionality at high concentrations used for the *in vivo* preparation protocol (see methods). High concentration of hPIs allows to evaluate if an increased interaction between hPIs ameliorates (or worsens) the quality of long-term hPIs cultures. To distinguish these concentrations, 25IEQ are named *Low-Density (LD) of hPIs* and 500IEQ as *High-Density (HD) of hPIs*. Low-Density and High-Density of hPIs in suspension in MIAMI medium, hPIs in suspension in GF medium, hPIs-embedded HYDROSAP in MIAMI medium and hPIs-embedded HYDROSAP in GF medium were compared at 28-days of culture. hPIs in suspension in MIAMI and GF media cultured for 28-days are considered as negative controls. All markers considered in previous analysis were studied in Low-Density and High-Density conditions (Figure 5).

Percentage of insulin positive β-cells increased (Figure 5A) in HYDROSAP in GF medium (41.82 ± 9.01%, HD of hPIs), compared to its LD counterpart in GF medium (26.45 ± 11.61%, ***p < 0.001), obtaining values similar to HYDROSAP in MIAMI medium at HD (45.29 ± 4.91%) and to freshly-isolated islets at T1 (45.84 ± 12.59%, Figure 2A). α-cells secerning glucagon (Figure 5B) significantly enhanced glucagon expression in HD condition in hPIs suspended in GF medium (32.98 ± 5.87%), in hPIs-embedded HYDROSAP in MIAMI medium (38.16 ± 7.68%) and in hPIs-embedded HYDROSAP in GF medium (36.98 ± 7.66%). As mentioned before, glucagon content is poorly conserved in HYDROSAP scaffold overtime. However, maintaining hPIs in HD for 28-days into HYDROSAP allowed to reach the value of freshly-isolated hPIs at T1 (28.76 ± 9.75%, Figure 2B). Similar results were achieved for endocrine cells (Figure 5C).

Thus, β-cells, α-cells and endocrine component showed increased values in HD cultures (especially for hPIs maintained in GF medium, hPIs-embedded HYDROSAP in MIAMI medium and also in GF medium). Despite the increased hPIs concentration, proliferative cells and apoptotic cells percentages inside islets remained unaffected (Figure 5D, E).

However, HD conditions negatively affected vWF and Fibroblast expression, that decreased drastically in most conditions with ***p < 0.001 (Figure 5F, G). On the contrary, the major components of ECM (laminin, collagen IV and collagen I) (Figure 5H–L) remained stable, except for collagen IV in HYDROSAP in GF medium that significantly increased its expression in HD condition (1.42 ± 0.87%, compared to 0.61 ± 0.20% at LD, *p < 0.05), and for Collagen I in HYDROSAP in MIAMI medium that drastically reduced its expression (0.45 ± 0.23% in HD compared to 1.21 ± 0.74% at LD, **p < 0.01).

In conclusion, HD cultures of hPIs in HYDROSAP up to 28 days showed an increased percentage of α-cells and β-cells, a controlled apoptosis, and a lower content of ECM components.

Encouraged by excellent *in vitro* results in term of preservation cellular components, we tested the efficacy of transplants of hPIs previously cultured *in vitro*. Inside HYDROSAP scaffold, β-cells and endocrine component were well-preserved up to 14 DIV and 28 DIV, if compared to standard hPIs cultures (hPIs suspensions at 1 day).

### 3.5 *In vivo* efficacy of hPIs pre-cultured in 3D bioscaffolds: Preliminary data

Effective functionality of hPIs maintained in culture for 2 and 4 weeks was investigated *in vivo* in a pilot study: hPIs were transplanted into sub-renal capsule of diabetic mice. The subcapsular space is considered an ideal site for pancreatic islets implantation in diabetic mice: indeed, pancreatic islets engraftment in the subrenal capsule site is a well-established protocol (Szot et al., 2007). Researchers reported that human islets transplantation into mice kidney cured diabetes in 75%–80% of recipients; while transplantation of murine islets yielded 100% success (Stokes et al., 2017). Animals in *vivo* experiments were divided into three groups: 1) six mice receiving freshly-isolated hPIs (control group), 2) six animals transplanted with hPIs pre-cultured in HYDROSAP for 2 weeks, and 3) six animals receiving hPIs pre-cultured in HYDROSAP for 4 weeks. In these experiments, only hPIs cultured in MIAMI medium were considered. Freshly-isolated hPIs were immediately transplanted into the sub-renal capsule; conversely, hPIs pre-cultured in HYDROSAP were maintained in culture for two or 4 weeks at HD of hPIs and mechanically dissociated from HYDROSAP immediately before transplantation. As best examples, here we report two mice of the control group and one animal implanted with hPIs (pre-cultured for 2 weeks) that responded positively to islets transplantation, approaching normoglycemic values (Supplementary Figure S4) and an optimal responsiveness to intraperitoneal glucose tolerance tests (Supplementary Figure S4), especially 15 days after transplantation (Supplementary Figure S4). Also, a progressive increase of body weight through the course of the study (Supplementary Figure S4) and the presence of transplanted functional hPIs at the implant site (Supplementary Figure S5) confirmed the success of engraftment in all three animals. The poor success of the overall *in vivo* transplants (3/18 mice) and, in particular, of positive controls (2/6 mice), led us to consider this study as preliminary. A refinement of implantation technique is required. Indeed, the injection technique could have partially destroyed cells, or the amount of engrafted hPIs was not enough to restore normoglycemia (Estil Les et al., 2018) and hPIs resulted unable to secrete a sufficient amount of insulin to reduce blood glucose concentration at physiological levels (Perteghella et al., 2017). Moreover, most animals developed a severe hyperglycemia after STZ injection with secondary complication, leading to a high mortality rate and a low success of engraftment (Supplementary Figure S6) (Deeds et al., 2011). For example, a decrease in weight in all mice receiving pre-cultured hPIs was detected (Supplementary Figure S6).

Being a pilot study, it is not possible to draw specific conclusions about the efficacy of our 3D treatment for hPIs for now, and further investigation is required. However, one animal recovered normoglycemia after transplantation with hPIs pre-cultured for 2 weeks, and such return to physiological glucose level is very likely related to the success of hPIs pre-culturing and engraftment.

## 4 Conclusion

In summary, we successfully developed a 3D culture system capable of preserving human pancreatic islet morphology and functionality up to 28-days *in vitro*. The scaffold formulation has been carefully selected to better recapitulate 3D issue-specific conditions. HYDROSAP scaffold favors long-term hPIs cultures, by supporting islets functionality, rounded hPIs morphology and islets diameter. In particular, hPIs embedded in HYDROSAP and cultured in MIAMI medium for 14-and 28-days have shown an excellent preservation of cellular content (β-cells producing insulin, endocrine cells, and proliferative cells), endothelial cells and ECM component (laminin, collagen I and collagen IV). Validation of efficacy of hPIs cultured *in vitro* was also tested in preliminary *in vivo* experiments, obtaining a partial restoration of physiological blood glucose level in a diabetic animal transplanted with hPIs maintained in culture for 14-days. Non-etheless, further pre-clinical studies need to follow, including the refinement of injection technique in the subrenal space and establishing the sufficient amount of hPIs to be injected for restoring normoglycemia. Still, our findings demonstrate the potential of a helpful approach for long-term cultures of hPIs, in terms of islets survival and functionality, and pave may the way for a significant improvement of the current clinical treatment of Type 1 Diabetes with hPIs transplants.

## Data Availability

The raw data supporting the conclusions of this article will be made available by the authors, without undue reservation.

## References

[B1] Abadpour S. Niemi E. M. Orrhult L. S. Hermanns C. De Vries R. Nogueira L. P. (2022). Adipose-derived stromal cells preserve pancreatic islet function in a transplantable 3D bioprinted scaffold. bioRxiv. 10.1101/2022.05.30.494035 PMC11469278 37781993

[B2] Alpert S. Hanahan D. Teitelman G. (1988). Hybrid insulin genes reveal a developmental lineage for pancreatic endocrine cells and imply a relationship with neurons. Cell 53, 295–308. 10.1016/0092-8674(88)90391-1 3282675

[B3] Angel P. M. Zambrzycki S. C. (2022). Predictive value of collagen in cancer. Adv. Cancer Res. 154, 15–45. 10.1016/bs.acr.2022.02.004 35459468

[B4] Bachul P. Borek P. Anteby R. Generette G. S. Basto L. Perea L. (2021). 207.2: Favorable 5-year follow up outcomes after islet transplantation in patients with type 1 diabetes mellitus at university of chicago. Transplantation 105, S3. 10.1097/01.tp.0000804288.00839.c4

[B5] Baldari S. Di Modugno F. Nisticò P. Toietta G. (2022). Strategies for efficient targeting of tumor collagen for cancer therapy. Cancers (Basel) 14, 4706. 10.3390/cancers14194706 36230627 PMC9563908

[B6] Banerjee I. (2020). “ECM-based scaffolds for pancreas bioengineering,” in Transplantation, bioengineering, and regeneration of the endocrine pancreas. Editors Orlando G. Piemonti L. Ricordi C. Stratta R. Gruessner R. (Academic Press), 2, 243–255. 10.1016/B978-0-12-814831-0.00017-8

[B7] Bertuzzi F. Colussi G. Lauterio A. De Carlis L. (2018a). Intramuscular islet allotransplantation in type 1 diabetes mellitus. Eur. Rev. Med. Pharmacol. Sci. 22, 1731–1736. 10.26355/eurrev_201803_14588 29630119

[B8] Bertuzzi F. De Carlis L. Marazzi M. Rampoldi A. G. Bonomo M. Antonioli B. (2018b). Long-term effect of islet transplantation on glycemic variability. Cell Transpl. 27, 840–846. 10.1177/0963689718763751 PMC6047271 29871516

[B9] Brandhorst D. Brandhorst H. Lee Layland S. Acreman S. Schenke-Layland K. Johnson P. R. V. (2022). Basement membrane proteins improve human islet survival in hypoxia: Implications for islet inflammation. Acta Biomater. 137, 92–102. 10.1016/j.actbio.2021.10.013 34653695

[B10] Brissova M. Powers A. C. (2008). Revascularization of transplanted islets: can it be improved? Diabetes 57, 2269–2271. 10.2337/db08-0814 18753672 PMC2518476

[B11] Caprini A. Silva D. Zanoni I. Cunha C. Volonte C. Vescovi A. (2013). A novel bioactive peptide: assessing its activity over murine neural stem cells and its potential for neural tissue engineering. N. Biotechnol. 30, 552–562. 10.1016/j.nbt.2013.03.005 23541699

[B12] Cayabyab F. Nih L. R. Yoshihara E. (2021). Advances in pancreatic islet transplantation sites for the treatment of diabetes. Front. Endocrinol. 12, 732431. 10.3389/fendo.2021.732431 PMC8473744 34589059

[B13] Chen J. Zou X. (2019). Self-assemble peptide biomaterials and their biomedical applications. Bioact. Mater 4, 120–131. 10.1016/j.bioactmat.2019.01.002 31667440 PMC6812166

[B14] Chen M. Bao L. Zhao M. Cao J. Zheng H. (2020). Progress in research on the role of FGF in the formation and treatment of corneal neovascularization. Front. Pharmacol. 11, 111. 10.3389/fphar.2020.00111 32158390 PMC7052042

[B15] Ciulla M. G. Pugliese R. Gelain F. (2022). Boosted cross-linking and characterization of high-performing self-assembling peptides. Nanomater. (Basel) 12, 320. 10.3390/nano12030320 PMC8838902 35159664

[B16] Cross S. E. Vaughan R. H. Willcox A. J. Mcbride A. J. Abraham A. A. Han B. (2017). Key matrix proteins within the pancreatic islet basement membrane are differentially digested during human islet isolation. Am. J. Transpl. 17, 451–461. 10.1111/ajt.13975 27456745

[B17] Deeds M. C. Anderson J. M. Armstrong A. S. Gastineau D. A. Hiddinga H. J. Jahangir A. (2011). Single dose streptozotocin-induced diabetes: considerations for study design in islet transplantation models. Lab. Anim. 45, 131–140. 10.1258/la.2010.010090 21478271 PMC3917305

[B18] Elizondo D. M. Brandy N. Z. D. Da Silva R. L. L. De Moura T. R. Ali J. Yang D. (2020). Pancreatic islets seeded in a novel bioscaffold forms an organoid to rescue insulin production and reverse hyperglycemia in models of type 1 diabetes. Sci. Rep. 10, 4362. 10.1038/s41598-020-60947-x 32152396 PMC7062832

[B19] Estil Les E. Téllez N. Nacher M. Montanya E. (2018). A model for human islet transplantation to immunodeficient streptozotocin-induced diabetic mice. Cell Transpl. 27, 1684–1691. 10.1177/0963689718801006 PMC6299193 30269527

[B20] Fernandez S. A. Champion K., S. Danielczak L. Gasparrini M. Paraskevas S. Leask R., L. (2022). Engineering vascularized islet macroencapsulation devices: An *in vitro* platform to study oxygen transport in perfused immobilized pancreatic beta cell cultures. Front. Bioeng. Biotechnol. 10, 884071. 10.3389/fbioe.2022.884071 35519615 PMC9061948

[B21] Gelain F. Cigognini D. Caprini A. Silva D. Colleoni B. Donega M. (2012). New bioactive motifs and their use in functionalized self-assembling peptides for NSC differentiation and neural tissue engineering. Nanoscale 4, 2946–2957. 10.1039/c2nr30220a 22476090

[B22] Gelain F. Luo Z. Rioult M. Zhang S. (2021). Self-assembling peptide scaffolds in the clinic. npj Regen. Med. 6, 9. 10.1038/s41536-020-00116-w 33597509 PMC7889856

[B23] Gelati M. Profico D. Projetti-Pensi M. Muzi G. Sgaravizzi G. Vescovi A. L. (2013). Culturing and expansion of "clinical grade" precursors cells from the fetal human central nervous system. Methods Mol. Biol. 1059, 65–77. 10.1007/978-1-62703-574-3_6 23934834

[B24] Guimarães C. F. Gasperini L. Marques A. P. Reis R. L. (2020). The stiffness of living tissues and its implications for tissue engineering. Nat. Rev. Mater. 5, 351–370. 10.1038/s41578-019-0169-1

[B25] Jiang L. Shen Y. Liu Y. Zhang L. Jiang W. (2022). Making human pancreatic islet organoids: Progresses on the cell origins, biomaterials and three-dimensional technologies. Theranostics 12, 1537–1556. 10.7150/thno.66670 35198056 PMC8825586

[B26] Kaido T. Yebra M. Cirulli V. Rhodes C. Diaferia G. Montgomery A. M. (2006). Impact of defined matrix interactions on insulin production by cultured human beta-cells: effect on insulin content, secretion, and gene transcription. Diabetes 55, 2723–2729. 10.2337/db06-0120 17003336

[B27] Katsarou A. Gudbjörnsdottir S. Rawshani A. Dabelea D. Bonifacio E. Anderson B. J. (2017). Type 1 diabetes mellitus. Nat. Rev. Dis. Prim. 3, 17016. 10.1038/nrdp.2017.16 28358037

[B28] Khiatah B. Qi M. Wu Y. Chen K. T. Perez R. Valiente L. (2019). Pancreatic human islets and insulin-producing cells derived from embryonic stem cells are rapidly identified by a newly developed Dithizone. Sci. Rep. 9, 9295. 10.1038/s41598-019-45678-y 31243300 PMC6594947

[B29] Kpeglo D. Hughes M. D. G. Dougan L. Haddrick M. Knowles M. A. Evans S. D. (2022). Modeling the mechanical stiffness of pancreatic ductal adenocarcinoma. Matrix Biol. Plus 14, 100109. 10.1016/j.mbplus.2022.100109 35399702 PMC8990173

[B30] Kumar N. Joisher H. Ganguly A. (2018). Polymeric scaffolds for pancreatic tissue engineering: A review. Rev. Diabet. Stud. 14, 334–353. 10.1900/RDS.2017.14.334 29590227 PMC6230446

[B31] Lee H. Y. Yea K. Kim J. Lee B. D. Chae Y. C. Kim H. S. (2008). Epidermal growth factor increases insulin secretion and lowers blood glucose in diabetic mice. J. Cell Mol. Med. 12, 1593–1604. 10.1111/j.1582-4934.2007.00169.x 18053093 PMC3918075

[B32] Lesavage B. L. Gilchrist A. E. Krajina B. A. Karlsson K. Smith A. R. Karagyozova K. (2022). Engineered extracellular matrices reveal stiffness-mediated chemoresistance in patient-derived pancreatic cancer organoids. bioRxiv. 10.1101/2022.04.22.488943 PMC13098013 38965405

[B33] Llacua L. A. Faas M. M. De Vos P. (2018). Extracellular matrix molecules and their potential contribution to the function of transplanted pancreatic islets. Diabetologia 61, 1261–1272. 10.1007/s00125-017-4524-8 29306997 PMC6449002

[B34] Marchini A. Gelain F. (2022). Synthetic scaffolds for 3D cell cultures and organoids: applications in regenerative medicine. Crit. Rev. Biotechnol. 42, 468–486. 10.1080/07388551.2021.1932716 34187261

[B35] Marchini A. Raspa A. Pugliese R. El Malek M. A. Pastori V. Lecchi M. (2019). Multifunctionalized hydrogels foster hNSC maturation in 3D cultures and neural regeneration in spinal cord injuries. Proc. Natl. Acad. Sci. U. S. A. 116, 7483–7492. 10.1073/pnas.1818392116 30923117 PMC6462084

[B36] Marchini A. Favoino C. Gelain F. (2020). Multi-functionalized self-assembling peptides as reproducible 3D cell culture systems enabling differentiation and survival of various human neural stem cell lines. Front. Neurosci. 14, 413. 10.3389/fnins.2020.00413 32431590 PMC7214803

[B37] Marin D. Marchesan S. (2022). Self-assembled peptide nanostructures for ECM biomimicry. Nanomater. (Basel) 12, 2147. 10.3390/nano12132147 PMC9268130 35807982

[B38] Matson J. B. Stupp S. I. (2012). Self-assembling peptide scaffolds for regenerative medicine. Chem. Commun. (Camb) 48, 26–33. 10.1039/c1cc15551b 22080255 PMC3355058

[B39] Parent A. V. Ashe S. Nair G. G. Li M. L. Chavez J. Liu J. S. (2022). Development of a scalable method to isolate subsets of stem cell-derived pancreatic islet cells. Stem Cell Rep. 17, 979–992. 10.1016/j.stemcr.2022.02.001 PMC9023773 35245441

[B40] Pellicciaro M. Vella I. Lanzoni G. Tisone G. Ricordi C. (2017). The greater omentum as a site for pancreatic islet transplantation. CellR4 Repair Replace. Regen. Reprogr. 5, e2410. PMC8025931 33834082

[B41] Perez-Basterrechea M. Esteban M. M. Alvarez-Viejo M. Fontanil T. Cal S. Sanchez Pitiot M. (2017). Fibroblasts accelerate islet revascularization and improve long-term graft survival in a mouse model of subcutaneous islet transplantation. PLoS One 12, e0180695. 10.1371/journal.pone.0180695 28672010 PMC5495486

[B42] Perez-Basterrechea M. Esteban M. M. Vega J. A. Obaya A. J. (2018). Tissue-engineering approaches in pancreatic islet transplantation. Biotechnol. Bioeng. 115, 3009–3029. 10.1002/bit.26821 30144310

[B43] Perteghella S. Vigani B. Mastracci L. Grillo F. Antonioli B. Galuzzi M. (2017). Stromal vascular fraction loaded silk fibroin mats effectively support the survival of diabetic mice after pancreatic islet transplantation. Macromol. Biosci. 17, 1700131. 10.1002/mabi.201700131 28691373

[B44] Perugini V. Flaherty S. M. Santin M. (2022). Development of scaffold-free vascularized pancreatic beta-islets *in vitro* models by the anchoring of cell lines to a bioligand-functionalized gelatine substrate. J. Mater. Sci. Mater. Med. 33, 37. 10.1007/s10856-022-06658-3 35403934 PMC9001567

[B45] Petrelli A. Carvello M. Vergani A. Lee K. M. Tezza S. Du M. (2011). IL-21 is an antitolerogenic cytokine of the late-phase alloimmune response. Diabetes 60, 3223–3234. 10.2337/db11-0880 22013017 PMC3219943

[B46] Pugliese R. Gelain F. (2017). Peptidic biomaterials: From self-assembling to regenerative medicine. Trends Biotechnol. 35, 145–158. 10.1016/j.tibtech.2016.09.004 27717599

[B47] Pugliese R. Fontana F. Marchini A. Gelain F. (2018a). Branched peptides integrate into self-assembled nanostructures and enhance biomechanics of peptidic hydrogels. Acta Biomater. 66, 258–271. 10.1016/j.actbio.2017.11.026 29128535

[B48] Pugliese R. Marchini A. Saracino G. A. Gelain F. (2018b). “Functionalization of self-assembling peptides for neural tissue engineering,” in Self-assembling biomaterials. Editors Azevedo H. S. da Silva R. M. P. (Woodhead Publishing), 475–493. 10.1016/B978-0-08-102015-9.00023-X

[B49] Pugliese R. Marchini A. Saracino G. a. A. Zuckermann R. N. Gelain F. (2018c). Cross-linked self-assembling peptide scaffolds. Nano Res. 11, 586–602. 10.1007/s12274-017-1834-6

[B50] Raspa A. Marchini A. Pugliese R. Mauri M. Maleki M. Vasita R. (2016). A biocompatibility study of new nanofibrous scaffolds for nervous system regeneration. Nanoscale 8, 253–265. 10.1039/c5nr03698d 26607419

[B51] Ricordi C. Lacy P. E. Finke E. H. Olack B. J. Scharp D. W. (1988). Automated method for isolation of human pancreatic islets. Diabetes 37, 413–420. 10.2337/diab.37.4.413 3288530

[B52] Ricordi C. Goldstein J. S. Balamurugan A. N. Szot G. L. Kin T. Liu C. (2016). National institutes of health-sponsored clinical islet transplantation consortium phase 3 trial: Manufacture of a complex cellular product at eight processing facilities. Diabetes 65, 3418–3428. 10.2337/db16-0234 27465220 PMC5079635

[B53] Riopel M. Wang R. (2014). Collagen matrix support of pancreatic islet survival and function. Front. Biosci. (Landmark Ed.) 19, 77–90. 10.2741/4196 24389173

[B54] Sakata N. Yoshimatsu G. Kodama S. (2020). The roles of collagen in islet transplantation. OBM Transplant. 04, 127. 10.21926/obm.transplant.2004127

[B55] Salg G. A. Giese N. A. Schenk M. Hüttner F. J. Felix K. Probst P. (2019). The emerging field of pancreatic tissue engineering: A systematic review and evidence map of scaffold materials and scaffolding techniques for insulin-secreting cells. J. Tissue Eng. 10, 204173141988470. 10.1177/2041731419884708 PMC6823987 31700597

[B56] Shapiro A. M. Lakey J. R. Ryan E. A. Korbutt G. S. Toth E. Warnock G. L. (2000). Islet transplantation in seven patients with type 1 diabetes mellitus using a glucocorticoid-free immunosuppressive regimen. N. Engl. J. Med. 343, 230–238. 10.1056/NEJM200007273430401 10911004

[B57] Song K. Yu Z. Zu X. Li G. Hu Z. Xue Y. (2022). Collagen remodeling along cancer progression providing a novel opportunity for cancer diagnosis and treatment. Int. J. Mol. Sci. 23, 10509. 10.3390/ijms231810509 36142424 PMC9502421

[B58] Stendahl J. C. Kaufman D. B. Stupp S. I. (2009). Extracellular matrix in pancreatic islets: relevance to scaffold design and transplantation. Cell Transpl. 18, 1–12. 10.3727/096368909788237195 PMC2724969 19476204

[B59] Stokes R. A. Cheng K. Lalwani A. Swarbrick M. M. Thomas H. E. Loudovaris T. (2017). Transplantation sites for human and murine islets. Diabetologia 60, 1961–1971. 10.1007/s00125-017-4362-8 28735354 PMC6448863

[B60] Szot G. L. Koudria P. Bluestone J. A. (2007). Transplantation of pancreatic islets into the kidney capsule of diabetic mice. J. Vis. Exp. 404, 404. 10.3791/404 PMC2566322 18989445

[B61] Takebe T. Wells J. M. (2019). Organoids by design. Science 364, 956–959. 10.1126/science.aaw7567 31171692 PMC8212787

[B62] Townsend S. E. Gannon M. (2019). Extracellular matrix-associated factors play critical roles in regulating pancreatic beta-cell proliferation and survival. Endocrinology 160, 1885–1894. 10.1210/en.2019-00206 31271410 PMC6656423

[B63] Vlahos A. E. Cober N. Sefton M. V. (2017). Modular tissue engineering for the vascularization of subcutaneously transplanted pancreatic islets. Proc. Natl. Acad. Sci. U. S. A. 114, 9337–9342. 10.1073/pnas.1619216114 28814629 PMC5584405

[B64] Wang W. Gu Y. Miyamoto M. Hori H. Nagata N. Balamurugan A. N. (2001). Effect of basic fibroblast growth factor on insulin secretion from microencapsulated pancreatic islets: An *in vitro* study. Cell Transplant. 10, 465–471. 10.3727/000000001783986521 11549073

[B65] Wieland F. C. Van Blitterswijk C. A. Van Apeldoorn A. Lapointe V. L. S. (2021). The functional importance of the cellular and extracellular composition of the islets of Langerhans. J. Immunol. Regen. Med. 13, 100048. 10.1016/j.regen.2021.100048

[B66] Xiong Y. Scerbo M. J. Seelig A. Volta F. O'brien N. Dicker A. (2020). Islet vascularization is regulated by primary endothelial cilia via VEGF-A-dependent signaling. eLife 9, e56914. 10.7554/eLife.56914 33200981 PMC7695455

[B67] Yin J. Meng H. Lin J. Ji W. Xu T. Liu H. (2022). Pancreatic islet organoids-on-a-chip: how far have we gone? J. Nanobiotechnol. 20, 308. 10.1186/s12951-022-01518-2 PMC9238112 35764957

[B68] Yu M. Agarwal D. Korutla L. May C. L. Wang W. Griffith N. N. (2020). Islet transplantation in the subcutaneous space achieves long-term euglycaemia in preclinical models of type 1 diabetes. Nat. Metab. 2, 1013–1020. 10.1038/s42255-020-0269-7 32895576 PMC7572844

[B69] Yuan Y. Cong C. Zhang J. Wei L. Li S. Chen Y. (2008). Self-assembling peptide nanofiber as potential substrates in islet transplantation. Transplant. Proc. 40, 2571–2574. 10.1016/j.transproceed.2008.08.017 18929804

[B70] Zhan L. Rao J. S. Sethia N. Slama M. Q. Han Z. Tobolt D. (2022). Pancreatic islet cryopreservation by vitrification achieves high viability, function, recovery and clinical scalability for transplantation. Nat. Med. 28, 798–808. 10.1038/s41591-022-01718-1 35288694 PMC9018423

[B71] Zhang W. Zhang S. Zhang W. Yue Y. Qian W. Wang Z. (2021). Matrix stiffness and its influence on pancreatic diseases. Biochim. Biophys. Acta (BBA) - Rev. Cancer 1876, 188583. 10.1016/j.bbcan.2021.188583 34139274

