# Supplementary material for: Long-term cultures of human pancreatic islets in self-assembling peptides hydrogels
Source: Front Bioeng Biotechnol. 2023 Feb 23;11:1105157. doi: 10.3389/fbioe.2023.1105157 (PMC9995881; doi:10.3389/fbioe.2023.1105157)
Supplement: Supplementary file 1 [file DataSheet1.docx]

Supplementary Material

# Supplementary Materials and Methods

## hPIs culture for *in vivo* study

## According to *in vitro* results, pre-cultured hPIs involved in *in vivo* study were cultured inside HYDROSAP (500IEQ) at high density for 2 or 4 weeks before transplantation. Immediately before surgery, pre-cultured hPIs for 2 and 4 weeks were recovered from HYDROSAP: hPIs was mechanically separated from HYDROSAP by pipetting in Hanks' Balanced Salt Solution (HBSS, Gibco). hPIs were finally pelleted by centrifuge (19g per 10min) and hand-picked using a p200 pipetman to reach a total number of 1500 IEQ per recipient.

## hPIs transplantation under kidney capsule of diabetic mice

Diabetes was induced by single intraperitoneal dose of Streptozotocin (STZ, 170 mg kg-1 in a volume of 10 ml/kg per mouse). Starting from 24/48 hours after STZ injection, blood was collected from the tail vein and the blood glucose levels measured with a glucometer. Animals were considered diabetic and ready for hPIs transplantation when they became hyperglycemic (non-fasting blood glucose level > 400 mg/dL). These diabetic recipient mice were prepared for surgery and anesthetized with 2% isoflurane. hPIs was pelleted by spontaneous precipitation into a gel-loading pipet tip, connected to 1mL syringe. Each animal received 1500 IEQ. A lumbar laparotomy of the skin and muscle were performed to access the kidney. The kidney was maintained outside the abdominal space and constantly hydrated with physiological solution to avoid drying of the capsule. An incision of 1–2 mm was made on kidney capsule and the gel-loading pipet tip was introduced to slowly dispense the microvolume of hPIs pellet. After injecting, the gel-loading pipet tip was removed, and the kidney capsule was cauterized to avoid hemorrhage and cell leakage. Muscles and skin layers were finally sutured. Tramadol (1-2 mg/kg) and/or Carprofen (5mg/kg) were subcutaneously administrated for acute pain in post-surgery period. Animals were caged 3-4 in autoclaved cages, with approximately 12-hour light and 12-hour dark cycle with pellets ad libitum. Mice were monitored and studied for 50 days post-surgery. At the end of experiment, mice were sacrificed by cervical dislocation. Engrafted kidney was excised, fixed with 4% PFA and incubated in sucrose solution (30% in PBS).

## Non-fasting blood glucose level (non-fasting BGL)

Glycemia level was evaluated with a glucometer starting from 24/48 hours after STZ administration, and every other day throughout the entire duration of the study. Blood was collected from tail vein. Animals were considered diabetic and ready for hPIs transplantation when they became hyperglycemic after two consecutive daily non-fasting BGL tests > 400mg/dL. After hPIs transplantation, normoglycemia was achieved when blood glucose levels scored values <200mg/dL. On the contrary, if two consecutive glucose levels were > 400mg/dL, islet engraftment was considered failed.

## Body weight measurement

Body weight measurements were collected every other day, until the terminal sacrifice.

## Intraperitoneal glucose tolerance test (IPGTT)

IPGTT were performed at 2 and 4 weeks after hPIs transplantation. Mice were fasted overnight before receiving a 2g/kg intraperitoneal glucose bolus (D-glucose, Sigma-Aldrich). Glucose levels were monitored at 0, 30, 60, 90, 120 minutes after injection.

## Immunohistochemical studies

After *in vivo* experimentation, engrafted kidney was excised, fixed with PFA 4%, incubated in sucrose 30% (in PBS) and cryopreserved in OCT. Tissues were cryo-sectioned at 30µm-thick slices via Cryostat (Histo-Line Laboratories). For immunofluorescence analyses, slices were washed in PBS, permeabilized with 0.3% Triton X-100 for 10 minutes at 4°C and treated with 10% normal goat serum (NGS, GIBCO) for 1 h at room temperature. Engrafted hPIs were stained with rabbit anti-insulin (1:300, ThermoFisher), following by secondary antibody goat anti-rabbit Cy3 (1:1000, Jackson). Cell nuclei were counterstained with HOECHST (Molecular Probes). Images were acquired at 40x magnification via Zeiss Microscope with Apotome System.

# Supplementary Figures


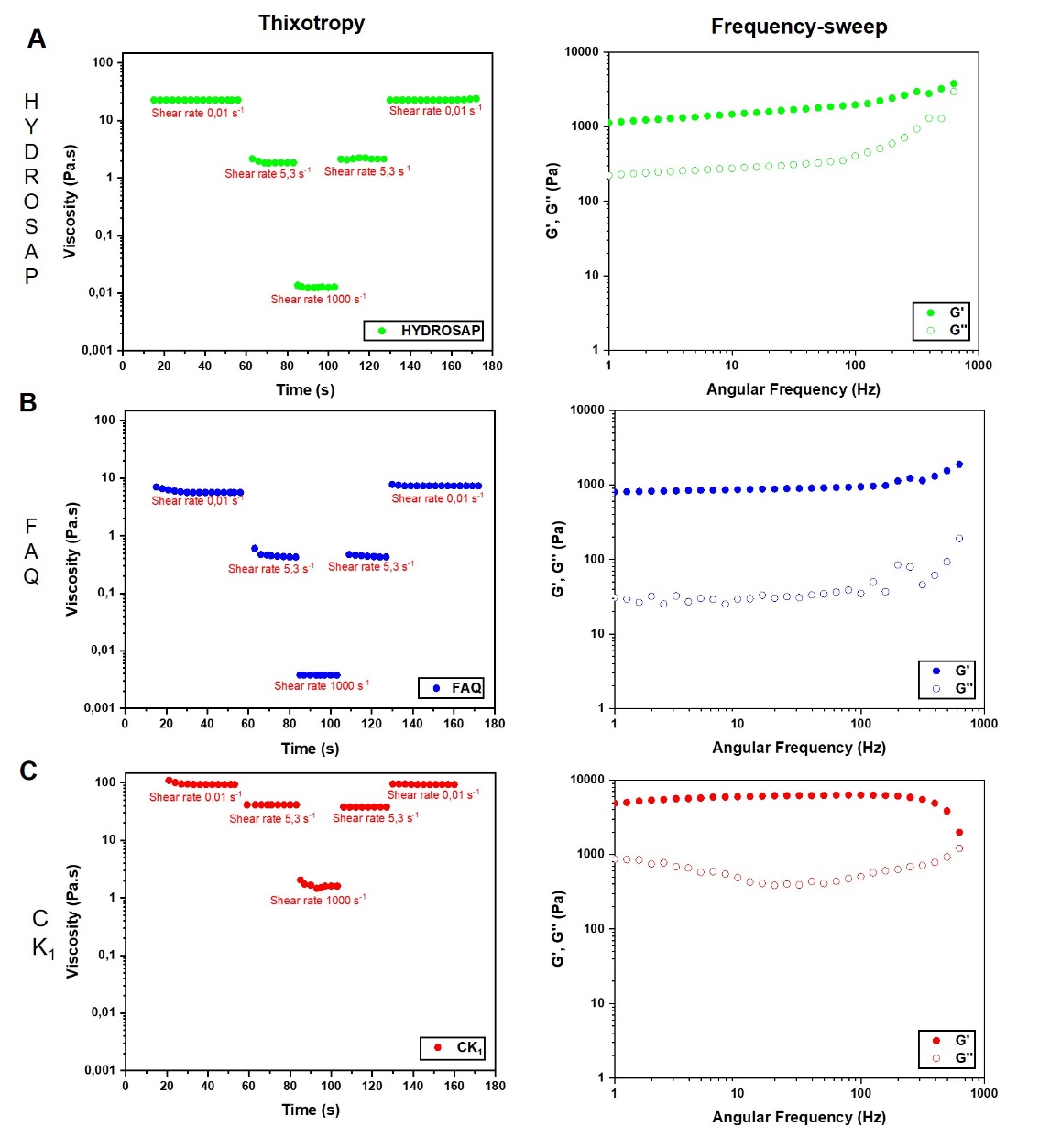


**Supplementary Figure 1.** Rheological measurements to study mechanical properties of (**A**) HYDROSAP, (**B**) FAQ, and (**C**) CK_1._ On the left, thixotropic behavior evaluation by applying alternated low and high shear-rate on materials. The shear-rates applied were, firstly, 0,01 s^-1^, followed by 5,3 s^-1^, and finally, the highest 1000 s^-1^. After completely recovery of values, all test confirmed the weak thixotropic and self-healing nature of these selected SAPs. On the right, frequency oscillatory experiments (0.1–100 Hz) at constant strain (1%) to evaluate the storage (G’) and loss (G”) moduli in the linear viscoelastic region. The measurements were recorded after a 15 h time-sweep test in presence of DPBS. The rheological profile revealed the typical predominant solid-like behavior of SAP hydrogels, showing a G’>G’’ and indicating their viscoelastic nature.


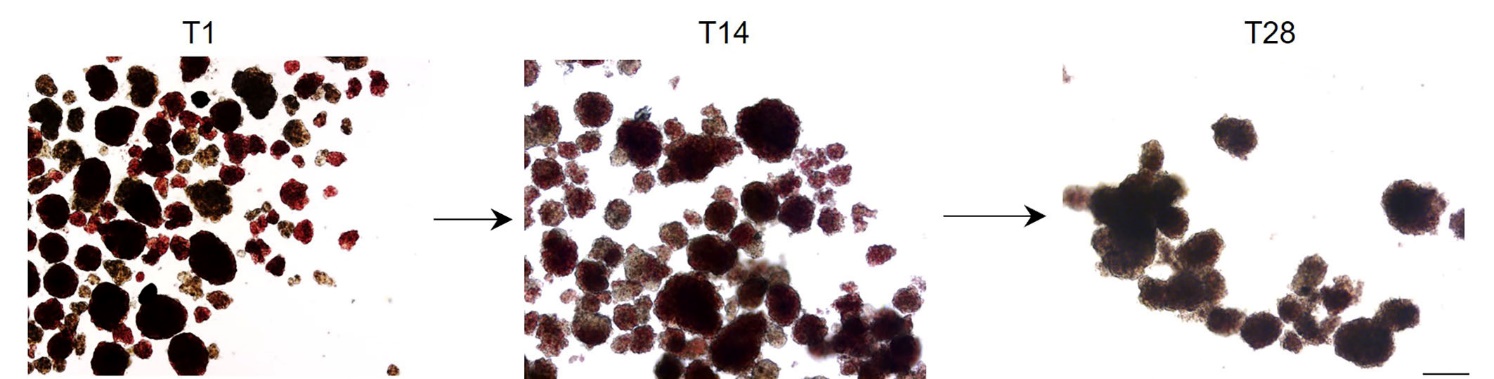


**Supplementary Figure 2.** Tracking hPIs in free-floating condition by Dithizone staining (islet quality, purity and morphology). At 1-day post-isolation (T1), hPIs appeared as distinct round cluster in reddish color, suggesting a high amount of zinc within β-cells and an optimal survival rate. After 14 (T14) and 28 (T28) days, hPIs clustered together, and reddish islets switched to brown color, revealing a loss of zinc ions. Indeed, Dithizone staining is a zinc chelating agent and stains functional pancreatic islets in reddish color. Zinc is an abundant element present within insulin granules of β-cells and is essential in maintaining structure and integrity of insulin molecules within β-cells. Scale bar, 100 µm.


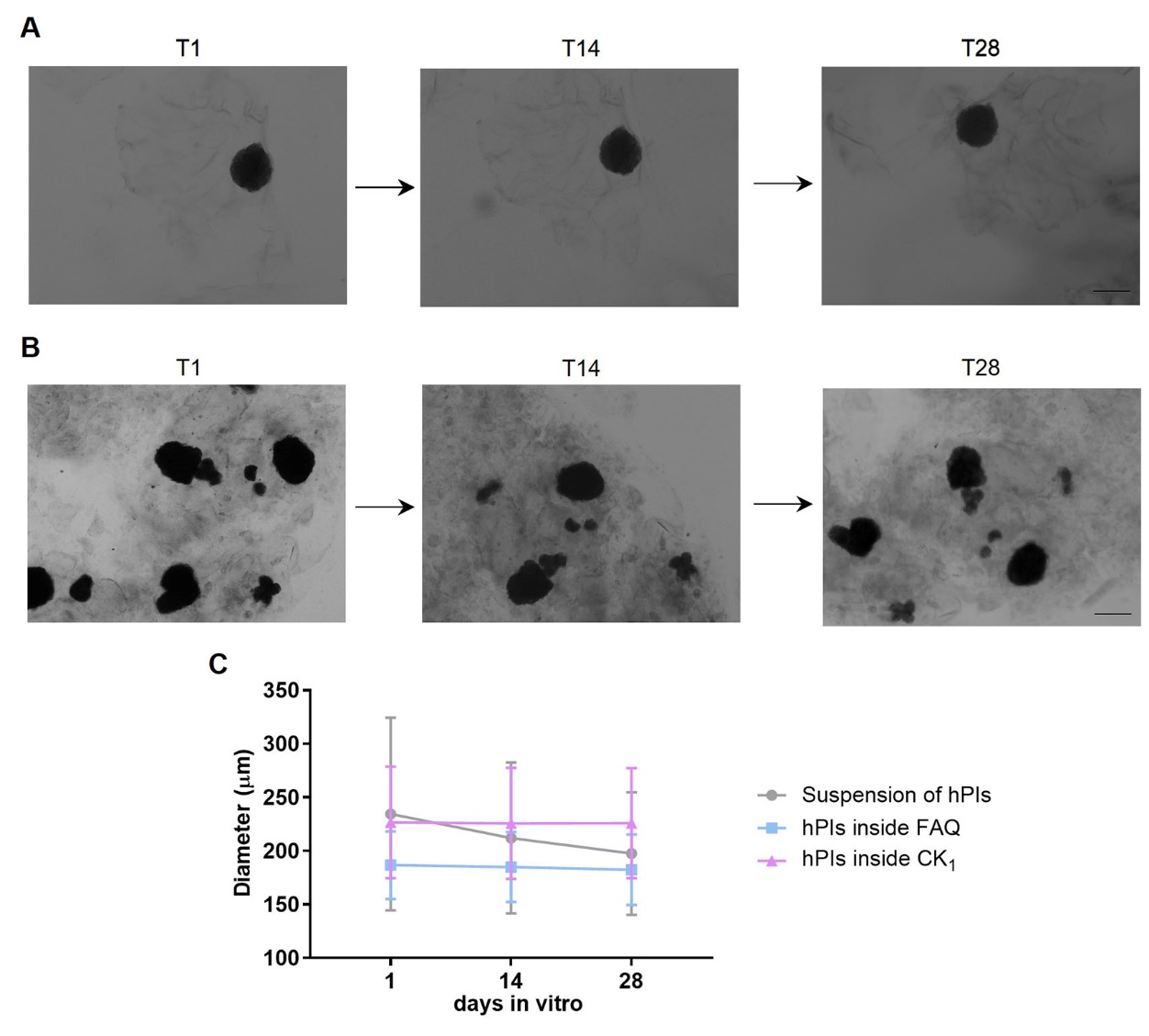


**Supplementary Figure 3.** Morphological evaluation of hPIs embedded inside FAQ (A) and CK_1_ (B). Islets morphology and integrity were tracked for 1-day post-isolation (T1), 14 days (T14) and 28 days (T28) via inverted microscope in brightfield: no morphological changes over weeks were observed. (C) Islets diameter for free-floating hPIs and hPIs embedded inside FAQ and CK_1_ hydrogels was measured over time till 28-day post-isolation. Graph shows mean ± SD of triplicate samples per each time point. Statistical difference between groups are represented in Supplementary Table 1. Scale bar, 100µm


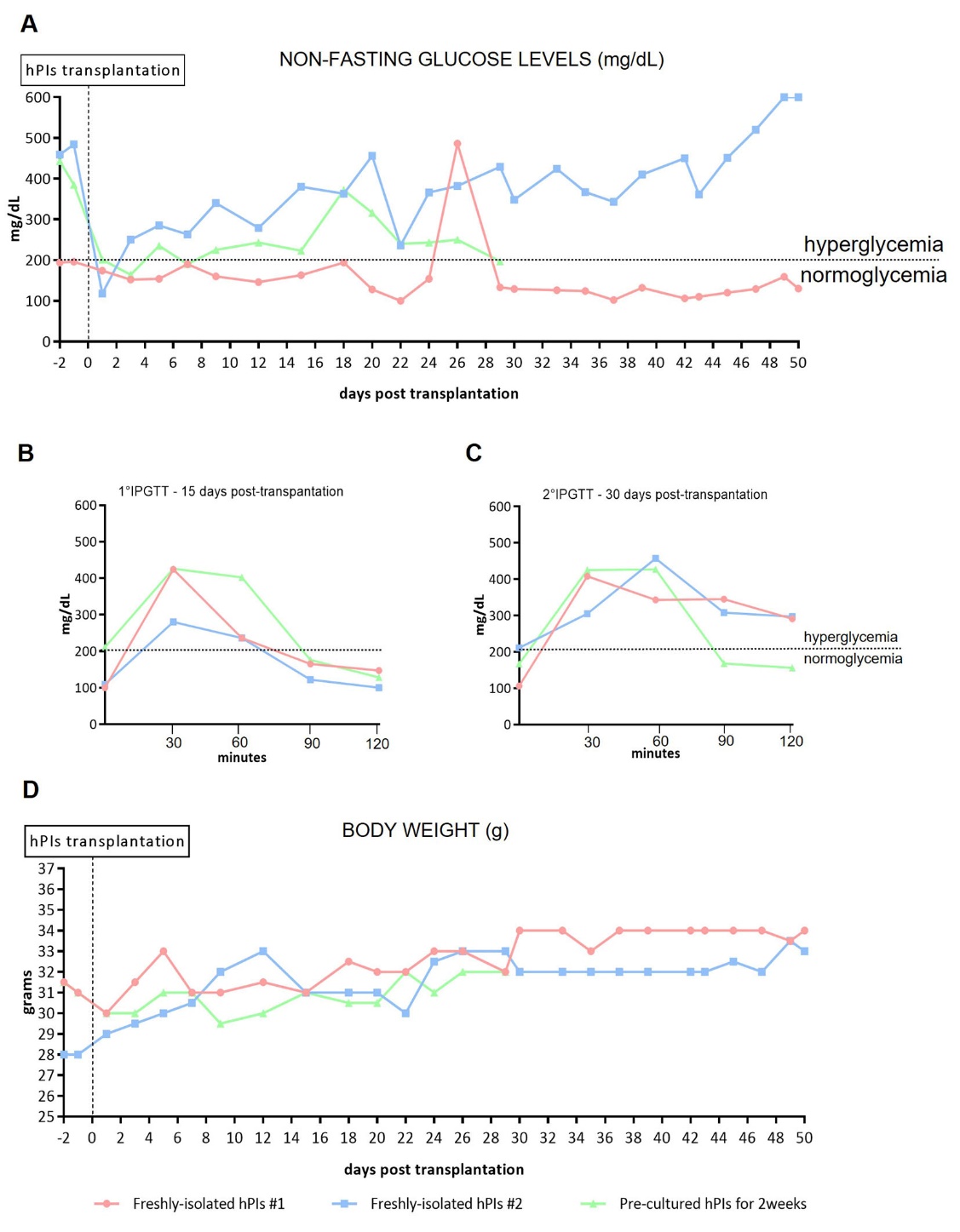


**Supplementary Figure 4.** Glycemia and body weight monitoring of the three animals that responded positively to islets transplantation: two mice transplanted with freshly-isolated hPIs (named #1 and #2), and one mouse receiving pre-cultured hPIs for 2 weeks. (**A**) Glycemia level measured through non-fasting glucose level from two days before hPIs transplantation to 50 days after surgery. For intraperitoneal glucose tolerance tests (IPGTT), blood glucose level was measured at (**B**) 15- and (**C**) 30-days post-surgery and glycemia level was recorded at 0, 30, 60, 90, 120 minutes after receiving intraperitoneal glucose bolus. (**D**) Time-tracking of body weight up to 50 days post-surgery. Day 0 is considered as the day of hPIs transplantation.


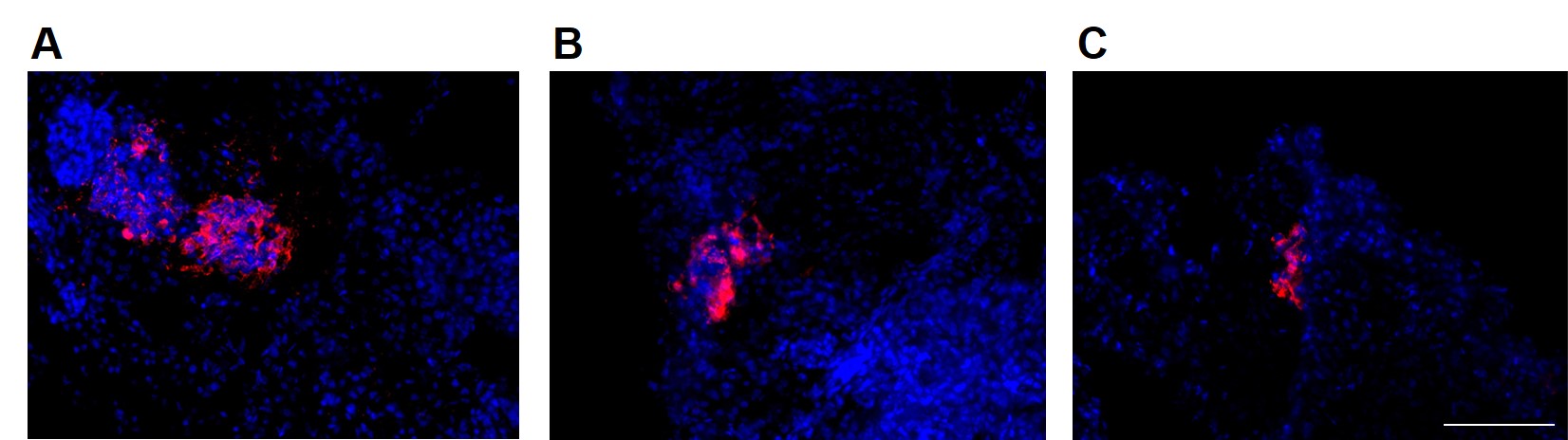


**Supplementary Figure 5.** Immunofluorescence images of implant site of (**A**) mouse #1 transplanted with freshly-isolated hPIs, (**B**) mouse #2 transplanted with freshly-isolated hPIs and (**C**) mouse transplanted with hPIs pre-cultured *in vitro* for 2weeks. Engrafted kidneys were cryo-sectioned and stained with insulin markers (in red) to verify the effective transplantation. Cell nuclei are labelled in blue. Scale bar, 100 µm.


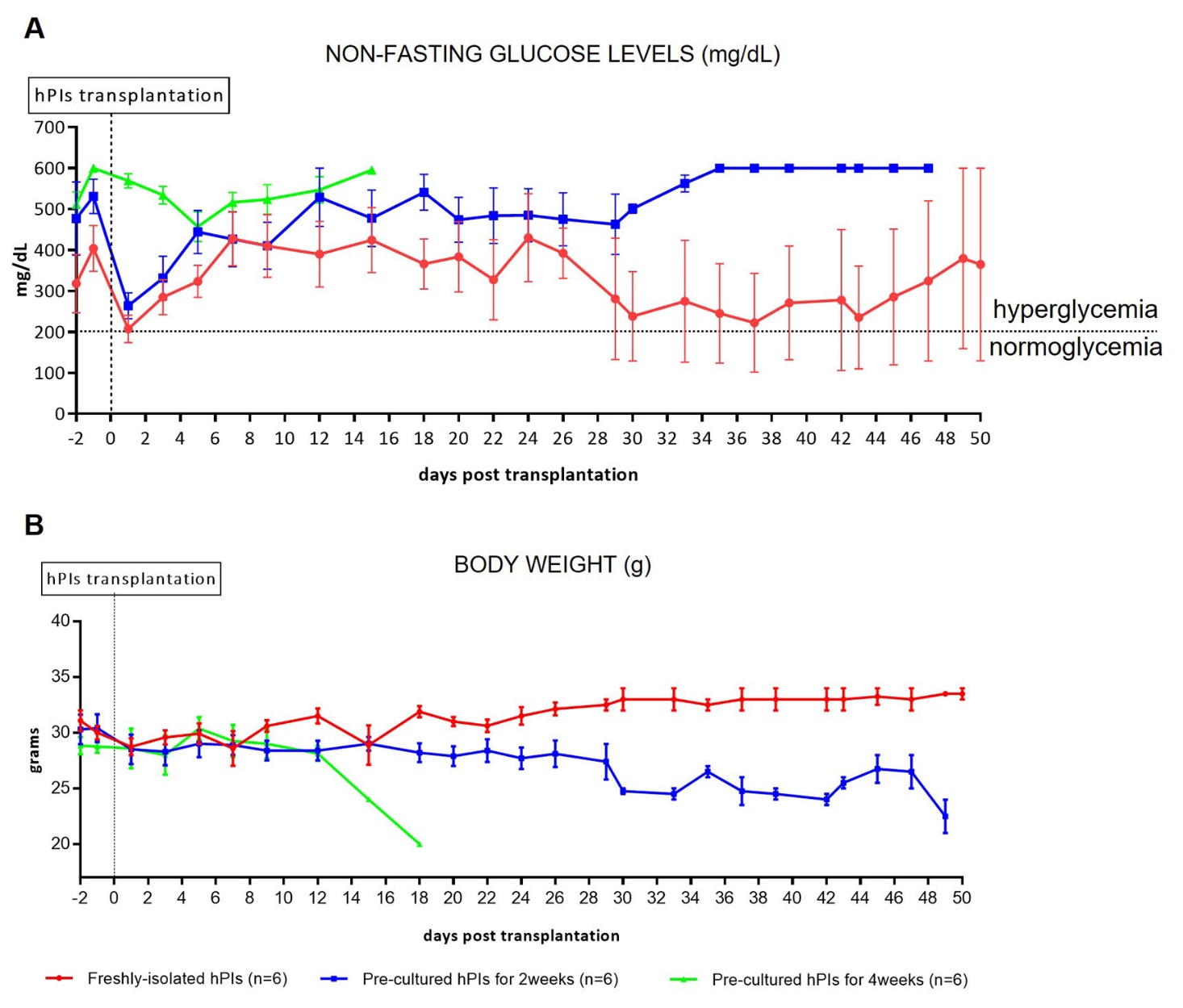


**Supplementary Figure 6.** Non-fasting glucose level and body weight monitoring of six mice transplanted with freshly-isolated hPIs, six mice receiving pre-cultured hPIs for 2 weeks, and six mice receiving pre-cultured hPIs for 4 weeks. (**A**) Glycemia was measured through non-fasting glucose level from two days before hPIs transplantation to 50 days after surgery and it revealed an overall severe hyperglycemia throughout the experimental timeframe. (**B**) Time-tracking of body weight up to 50 days post-surgery revealed a decrease in weight in pre-cultured hPI-treated mice, especially for mice receiving 4 weeks pre-cultured hPIs. Day 0 is the day of hPIs transplantation. Results are represented as mean ± SEM

**Supplementary Tables**

**Supplementary Table 1. Statistical analysis of islets diameter for free-floating hPIs and hPIs embedded inside HYDROSAP, FAQ and CK_1_ hydrogels.** Two-way ANOVA followed by Tukey post-test was performed for each condition after 1-day, 14-days, and 28-days post-isolation.

|  | T1 | T14 | T28 |
| --- | --- | --- | --- |
| Suspension of hPIs vs. hPIs inside HYDROSAP | ns | *** | *** |
| Suspension of hPIs vs. hPIs inside FAQ | *** | * | ns |
| Suspension of hPIs vs. hPIs inside CK_1_ | ns | ns | * |
| hPIs inside HYDROSAP vs. hPIs inside FAQ | *** | *** | *** |
| hPIs inside HYDROSAP vs. hPIs inside CK_1_ | ** | * | ns |
| hPIs inside FAQ vs. hPIs inside CK_1_ | *** | *** | *** |

**p < 0.05; **p < 0.01; ***p < 0.001; ns = not significant*

**Supplementary Table 2. Statistical analysis of graphs represented in Figure 2 concerning insulin, glucagon, chromogranin, proliferative cells, and apoptotic cells markers**. Two-way ANOVA followed by Tukey post-test was performed for hPIs cultured for 14-days in HYDROSAP, FAQ and CK_1_ scaffolds, or plated in suspension in MIAMI medium or GF medium for 1-day and 14-days.

| **INSULIN** | **P value** |
| --- | --- |
| MIAMI MEDIUM:SUSPENSION T1 vs. MIAMI MEDIUM:SUSPENSION T14 | *** |
| MIAMI MEDIUM:SUSPENSION T1 vs. MIAMI MEDIUM:FAQ T14 | * |
| MIAMI MEDIUM:SUSPENSION T1 vs. MIAMI MEDIUM:CK_1_ T14 | ** |
| MIAMI MEDIUM:SUSPENSION T1 vs. GF MEDIUM:SUSPENSION T14 | *** |
| MIAMI MEDIUM:SUSPENSION T1 vs. GF MEDIUM:HYDROSAP T14 | * |
| MIAMI MEDIUM:SUSPENSION T1 vs. GF MEDIUM:FAQ T14 | ** |
| MIAMI MEDIUM:SUSPENSION T1 vs. GF MEDIUM:CK_1_ T14 | *** |
| MIAMI MEDIUM:SUSPENSION T14 vs. MIAMI MEDIUM:HYDROSAP T14 | ** |
| MIAMI MEDIUM:SUSPENSION T14 vs. GF MEDIUM:SUSPENSION T1 | *** |
| MIAMI MEDIUM:SUSPENSION T14 vs. GF MEDIUM:HYDROSAP T14 | * |
| GF MEDIUM:SUSPENSION T1 vs. GF MEDIUM:CK_1_ T14 | * |

| **GLUCAGON** | **P value** |
| --- | --- |
| MIAMI MEDIUM:SUSPENSION T1 vs. MIAMI MEDIUM:SUSPENSION T14 | *** |
| MIAMI MEDIUM:SUSPENSION T1 vs. MIAMI MEDIUM:HYDROSAP T14 | *** |
| MIAMI MEDIUM:SUSPENSION T1 vs. MIAMI MEDIUM:FAQ T14 | *** |
| MIAMI MEDIUM:SUSPENSION T1 vs. MIAMI MEDIUM:CK_1_ T14 | *** |
| MIAMI MEDIUM:SUSPENSION T1 vs. GF MEDIUM:SUSPENSION T14 | *** |
| MIAMI MEDIUM:SUSPENSION T1 vs. GF MEDIUM:HYDROSAP T14 | *** |
| MIAMI MEDIUM:SUSPENSION T1 vs. GF MEDIUM:FAQ T14 | *** |
| MIAMI MEDIUM:SUSPENSION T1 vs. GF MEDIUM:CK_1_ T14 | *** |
| MIAMI MEDIUM:SUSPENSION T14 vs. MIAMI MEDIUM:HYDROSAP T14 | ** |
| MIAMI MEDIUM:SUSPENSION T14 vs. GF MEDIUM:SUSPENSION T1 | *** |
| MIAMI MEDIUM:SUSPENSION T14 vs. GF MEDIUM:HYDROSAP T14 | *** |
| MIAMI MEDIUM:HYDROSAP T14 vs. MIAMI MEDIUM:CK_1_ T14 | * |
| MIAMI MEDIUM:HYDROSAP T14 vs. GF MEDIUM:SUSPENSION T1 | *** |
| MIAMI MEDIUM:FAQ T14 vs. GF MEDIUM:SUSPENSION T1 | *** |
| MIAMI MEDIUM:CK_1_ T14 vs. GF MEDIUM:SUSPENSION T1 | *** |
| MIAMI MEDIUM:CK_1_ T14 vs. GF MEDIUM:HYDROSAP T14 | ** |
| GF MEDIUM:SUSPENSION T1 vs. GF MEDIUM:SUSPENSION T14 | *** |
| GF MEDIUM:SUSPENSION T1 vs. GF MEDIUM:HYDROSAP T14 | *** |
| GF MEDIUM:SUSPENSION T1 vs. GF MEDIUM:FAQ T14 | *** |
| GF MEDIUM:SUSPENSION T1 vs. GF MEDIUM:CK_1_ T14 | *** |
| GF MEDIUM:SUSPENSION T14 vs. GF MEDIUM:HYDROSAP T14 | * |

| **CHROMOGRANIN** | **P value** |
| --- | --- |
| MIAMI MEDIUM:SUSPENSION T1 vs. MIAMI MEDIUM:SUSPENSION T14 | *** |
| MIAMI MEDIUM:SUSPENSION T1 vs. MIAMI MEDIUM:FAQ T14 | * |
| MIAMI MEDIUM:SUSPENSION T1 vs. MIAMI MEDIUM:CK_1_ T14 | * |
| MIAMI MEDIUM:SUSPENSION T1 vs. GF MEDIUM:SUSPENSION T14 | * |
| MIAMI MEDIUM:SUSPENSION T1 vs. GF MEDIUM:FAQ T14 | * |
| MIAMI MEDIUM:SUSPENSION T1 vs. GF MEDIUM:CK_1_ T14 | ** |
| MIAMI MEDIUM:SUSPENSION T14 vs. GF MEDIUM:SUSPENSION T1 | *** |
| MIAMI MEDIUM:FAQ T14 vs. GF MEDIUM:SUSPENSION T1 | * |
| GF MEDIUM:SUSPENSION T1 vs. GF MEDIUM:CK_1_ T14 | ** |

| **Ki67** | **P value** |
| --- | --- |
| MIAMI MEDIUM:SUSPENSION T14 vs. MIAMI MEDIUM:HYDROSAP T14 | * |

| **TUNEL ASSAY** | **P value** |
| --- | --- |
| MIAMI MEDIUM:SUSPENSION T1 vs. MIAMI MEDIUM:SUSPENSION T14 | ** |
| MIAMI MEDIUM:SUSPENSION T1 vs. MIAMI MEDIUM:CK_1_ T14 | ** |
| MIAMI MEDIUM:SUSPENSION T14 vs. GF MEDIUM:SUSPENSION T1 | ** |
| MIAMI MEDIUM:CK_1_ T14 vs. GF MEDIUM:SUSPENSION T1 | *** |

**p < 0.05; **p < 0.01; ***p < 0.001.*

**Supplementary Table 3. Statistical analysis of graphs represented in Figure 3 concerning vWF, Fibroblast, Collagen IV and Collagen I markers**. Two-way ANOVA followed by Tukey post-test was performed for hPIs cultured for 14-days in HYDROSAP, FAQ and CK_1_ scaffolds, or plated in suspension in MIAMI medium or GF medium for 1-day and 14-days.

| **vWF** | **P value** |
| --- | --- |
| MIAMI MEDIUM:SUSPENSION T1 vs. GF MEDIUM:HYDROSAP T14 | *** |
| MIAMI MEDIUM:SUSPENSION T14 vs. MIAMI MEDIUM:HYDROSAP T14 | ** |
| MIAMI MEDIUM:SUSPENSION T14 vs. MIAMI MEDIUM:CK_1_ T14 | *** |
| MIAMI MEDIUM:SUSPENSION T14 vs. GF MEDIUM:HYDROSAP T14 | *** |
| MIAMI MEDIUM:HYDROSAP T14 vs. GF MEDIUM:HYDROSAP T14 | ** |
| MIAMI MEDIUM:FAQ T14 vs. GF MEDIUM:HYDROSAP T14 | *** |
| MIAMI MEDIUM:CK_1_ T14 vs. GF MEDIUM:HYDROSAP T14 | ** |
| GF MEDIUM:SUSPENSION T1 vs. GF MEDIUM:HYDROSAP T14 | *** |
| GF MEDIUM:SUSPENSION T14 vs. GF MEDIUM:HYDROSAP T14 | *** |
| GF MEDIUM:HYDROSAP T14 vs. GF MEDIUM:FAQ T14 | *** |
| GF MEDIUM:HYDROSAP T14 vs. GF MEDIUM:CK_1_ T14 | *** |

| **FIBROBLAST** | **P value** |
| --- | --- |
| MIAMI MEDIUM:SUSPENSION T1 vs. GF MEDIUM:SUSPENSION T1 | ** |
| MIAMI MEDIUM:SUSPENSION T1 vs. GF MEDIUM:HYDROSAP T14 | *** |
| MIAMI MEDIUM:SUSPENSION T14 vs. MIAMI MEDIUM:HYDROSAP T14 | *** |
| MIAMI MEDIUM:SUSPENSION T14 vs. GF MEDIUM:SUSPENSION T1 | *** |
| MIAMI MEDIUM:SUSPENSION T14 vs. GF MEDIUM:HYDROSAP T14 | *** |
| MIAMI MEDIUM:SUSPENSION T14 vs. GF MEDIUM:FAQ T14 | *** |
| MIAMI MEDIUM:HYDROSAP T14 vs. MIAMI MEDIUM:CK_1_ T14 | *** |
| MIAMI MEDIUM:HYDROSAP T14 vs. GF MEDIUM:SUSPENSION T14 | * |
| MIAMI MEDIUM:HYDROSAP T14 vs. GF MEDIUM:CK_1_ T14 | * |
| MIAMI MEDIUM:FAQ T14 vs. GF MEDIUM:SUSPENSION T1 | ** |
| MIAMI MEDIUM:FAQ T14 vs. GF MEDIUM:HYDROSAP T14 | *** |
| MIAMI MEDIUM:CK_1_ T14 vs. GF MEDIUM:SUSPENSION T1 | *** |
| MIAMI MEDIUM:CK_1_ T14 vs. GF MEDIUM:HYDROSAP T14 | *** |
| MIAMI MEDIUM:CK_1_ T14 vs. GF MEDIUM:FAQ T14 | * |
| GF MEDIUM:SUSPENSION T1 vs. GF MEDIUM:SUSPENSION T14 | *** |
| GF MEDIUM:SUSPENSION T1 vs. GF MEDIUM:CK_1_ T14 | *** |
| GF MEDIUM:SUSPENSION T14 vs. GF MEDIUM:HYDROSAP T14 | *** |
| GF MEDIUM:HYDROSAP T14 vs. GF MEDIUM:CK_1_ T14 | *** |

| **COLLAGEN IV** | **P value** |
| --- | --- |
| MIAMI MEDIUM:SUSPENSION T1 vs. GF MEDIUM:CK_1_ T14 | *** |
| MIAMI MEDIUM:SUSPENSION T14 vs. MIAMI MEDIUM:CK_1_ T14 | ** |
| MIAMI MEDIUM:SUSPENSION T14 vs. GF MEDIUM:HYDROSAP T14 | * |
| MIAMI MEDIUM:SUSPENSION T14 vs. GF MEDIUM:CK_1_ T14 | *** |
| MIAMI MEDIUM:FAQ T14 vs. GF MEDIUM:CK_1_ T14 | * |
| GF MEDIUM:SUSPENSION T1 vs. GF MEDIUM:CK_1_ T14 | * |
| GF MEDIUM:SUSPENSION T14 vs. GF MEDIUM:CK_1_ T14 | ** |
| GF MEDIUM:FAQ T14 vs. GF MEDIUM:CK_1_ T14 | ** |

| **COLLAGEN I** | **P value** |
| --- | --- |
| MIAMI MEDIUM:SUSPENSION T1 vs. GF MEDIUM:CK_1_ T14 | * |

**p < 0.05; **p < 0.01; ***p < 0.001.*

**Supplementary Table 4. Statistical analysis of graphs represented in Figure 4 concerning Insulin, Glucagon, Chromogranin, Ki67, apoptotic cells, vWF, Fibroblast, Laminin, Collagen IV and Collagen I markers**. Two-way ANOVA followed by Tukey post-test was performed for hPIs cultured for 14-days and 28-days in HYDROSAP scaffold or plated in suspension in MIAMI medium or GF medium for 1-day, 14-days, and 28-days.

| **INSULIN** | **P value** |
| --- | --- |
| MIAMI MEDIUM:SUSPENSION T1 vs. MIAMI MEDIUM:SUSPENSION T14 | *** |
| MIAMI MEDIUM:SUSPENSION T1 vs. MIAMI MEDIUM:SUSPENSION T28 | *** |
| MIAMI MEDIUM:SUSPENSION T1 vs. GF MEDIUM:SUSPENSION T14 | *** |
| MIAMI MEDIUM:SUSPENSION T1 vs. GF MEDIUM:SUSPENSION T28 | ** |
| MIAMI MEDIUM:SUSPENSION T1 vs. GF MEDIUM:HYDROSAP T14 | ** |
| MIAMI MEDIUM:SUSPENSION T1 vs. GF MEDIUM:HYDROSAP T28 | *** |
| MIAMI MEDIUM:SUSPENSION T14 vs. MIAMI MEDIUM:HYDROSAP T14 | ** |
| MIAMI MEDIUM:SUSPENSION T14 vs. MIAMI MEDIUM:HYDROSAP T28 | *** |
| MIAMI MEDIUM:SUSPENSION T14 vs. GF MEDIUM:SUSPENSION T1 | *** |
| MIAMI MEDIUM:SUSPENSION T14 vs. GF MEDIUM:HYDROSAPT14 | ** |
| MIAMI MEDIUM:SUSPENSION T14 vs. GF MEDIUM:HYDROSAP T28 | ** |
| MIAMI MEDIUM:SUSPENSION T28 vs. GF MEDIUM:SUSPENSION T1 | * |
| MIAMI MEDIUM:HYDROSAP T28 vs. GF MEDIUM:SUSPENSION T14 | * |
| GF MEDIUM:SUSPENSION T1 vs. GF MEDIUM:SUSPENSION T14 | * |

| **GLUCAGON** | **P value** |
| --- | --- |
| MIAMI MEDIUM:SUSPENSION T1 vs. MIAMI MEDIUM:SUSPENSION T14 | *** |
| MIAMI MEDIUM:SUSPENSION T1 vs. MIAMI MEDIUM:SUSPENSION T28 | * |
| MIAMI MEDIUM:SUSPENSION T1 vs. MIAMI MEDIUM:HYDROSAP T14 | *** |
| MIAMI MEDIUM:SUSPENSION T1 vs. GF MEDIUM:SUSPENSION T14 | *** |
| MIAMI MEDIUM:SUSPENSION T1 vs. GF MEDIUM:SUSPENSION T28 | ** |
| MIAMI MEDIUM:SUSPENSION T1 vs. GF MEDIUM:HYDROSAP T14 | ** |
| MIAMI MEDIUM:SUSPENSION T14 vs. MIAMI MEDIUM:SUSPENSION T28 | *** |
| MIAMI MEDIUM:SUSPENSION T14 vs. MIAMI MEDIUM:HYDROSAP T14 | * |
| MIAMI MEDIUM:SUSPENSION T14 vs. MIAMI MEDIUM:HYDROSAP T28 | *** |
| MIAMI MEDIUM:SUSPENSION T14 vs. GF MEDIUM:SUSPENSION T1 | *** |
| MIAMI MEDIUM:SUSPENSION T14 vs. GF MEDIUM:SUSPENSION T28 | ** |
| MIAMI MEDIUM:SUSPENSION T14 vs. GF MEDIUM:HYDROSAP T14 | ** |
| MIAMI MEDIUM:SUSPENSION T14 vs. GF MEDIUM:HYDROSAP T28 | *** |
| MIAMI MEDIUM:SUSPENSION T28 vs. GF MEDIUM:SUSPENSION T1 | * |
| MIAMI MEDIUM:SUSPENSION T28 vs. GF MEDIUM:SUSPENSION T14 | ** |
| MIAMI MEDIUM:HYDROSAP T14 vs. MIAMI MEDIUM:HYDROSAP T28 | * |
| MIAMI MEDIUM:HYDROSAP T14 vs. GF MEDIUM:SUSPENSION T1 | *** |
| MIAMI MEDIUM:HYDROSAP T28 vs. GF MEDIUM:SUSPENSION T14 | *** |
| GF MEDIUM:SUSPENSION T1 vs. GF MEDIUM:SUSPENSION T14 | *** |
| GF MEDIUM:SUSPENSION T1 vs. GF MEDIUM:SUSPENSION T28 | ** |
| GF MEDIUM:SUSPENSION T1 vs. GF MEDIUM:HYDROSAP T14 | ** |
| GF MEDIUM:SUSPENSION T14 vs. GF MEDIUM:HYDROSAP T28 | *** |

| **CHROMOGRANIN** | **P value** |
| --- | --- |
| MIAMI MEDIUM:SUSPENSION T1 vs. MIAMI MEDIUM:SUSPENSION T14 | *** |
| MIAMI MEDIUM:SUSPENSION T1 vs. MIAMI MEDIUM:SUSPENSION T28 | * |
| MIAMI MEDIUM:SUSPENSION T1 vs. GF MEDIUM:SUSPENSION T14 | ** |
| MIAMI MEDIUM:SUSPENSION T1 vs. GF MEDIUM:SUSPENSION T28 | *** |
| MIAMI MEDIUM:SUSPENSION T1 vs. GF MEDIUM:HYDROSAP T28 | ** |
| MIAMI MEDIUM:SUSPENSION T14 vs. MIAMI MEDIUM:HYDROSAP T14 | * |
| MIAMI MEDIUM:SUSPENSION T14 vs. MIAMI MEDIUM:HYDROSAP T28 | * |
| MIAMI MEDIUM:SUSPENSION T14 vs. GF MEDIUM:SUSPENSION T1 | *** |
| MIAMI MEDIUM:SUSPENSION T14 vs. GF MEDIUM:HYDROSAP T14 | * |
| GF MEDIUM:SUSPENSION T1 vs. GF MEDIUM:SUSPENSION T14 | * |
| GF MEDIUM:SUSPENSION T1 vs. GF MEDIUM:SUSPENSION T28 | ** |
| GF MEDIUM:SUSPENSION T1 vs. GF MEDIUM:HYDROSAP T28 | * |

| **Ki67** | **P value** |
| --- | --- |
| MIAMI MEDIUM:SUSPENSION T14 vs. MIAMI MEDIUM:HYDROSAP T14 | * |
| MIAMI MEDIUM:SUSPENSION T28 vs. MIAMI MEDIUM:HYDROSAP T14 | * |
| MIAMI MEDIUM:SUSPENSION T28 vs. GF MEDIUM:HYDROSAP T14 | * |
| MIAMI MEDIUM:HYDROSAP T14 vs. MIAMI MEDIUM:HYDROSAP T28 | * |
| MIAMI MEDIUM:HYDROSAP T14 vs. GF MEDIUM:SUSPENSION T28 | *** |
| MIAMI MEDIUM:HYDROSAP T28 vs. GF MEDIUM:HYDROSAP T14 | * |
| GF MEDIUM:SUSPENSION T1 vs. GF MEDIUM:SUSPENSION T28 | ** |
| GF MEDIUM:SUSPENSION T14 vs. GF MEDIUM:SUSPENSION T28 | ** |
| GF MEDIUM:SUSPENSION T28 vs. GF MEDIUM:HYDROSAP T14 | *** |

| **TUNEL ASSAY** | **P value** |
| --- | --- |
| MIAMI MEDIUM:SUSPENSION T1 vs. MIAMI MEDIUM:SUSPENSION T14 | *** |
| MIAMI MEDIUM:SUSPENSION T1 vs. MIAMI MEDIUM:HYDROSAP T14 | * |
| MIAMI MEDIUM:SUSPENSION T1 vs. GF MEDIUM:SUSPENSION T14 | * |
| MIAMI MEDIUM:SUSPENSION T1 vs. GF MEDIUM:HYDROSAP T28 | * |
| MIAMI MEDIUM:SUSPENSION T14 vs. MIAMI MEDIUM:HYDROSAP T28 | ** |
| MIAMI MEDIUM:SUSPENSION T14 vs. GF MEDIUM:SUSPENSION T1 | *** |
| MIAMI MEDIUM:SUSPENSION T14 vs. GF MEDIUM:SUSPENSION T28 | * |
| MIAMI MEDIUM:SUSPENSION T28 vs. GF MEDIUM:SUSPENSION T1 | * |
| MIAMI MEDIUM:HYDROSAP T14 vs. GF MEDIUM:SUSPENSION T1 | ** |
| GF MEDIUM:SUSPENSION T1 vs. GF MEDIUM:SUSPENSION T14 | ** |
| GF MEDIUM:SUSPENSION T1 vs. GF MEDIUM:HYDROSAP T14 | * |
| GF MEDIUM:SUSPENSION T1 vs. GF MEDIUM:HYDROSAP T28 | ** |

| **vWF** | **P value** |
| --- | --- |
| MIAMI MEDIUM:SUSPENSION T1 vs. GF MEDIUM:HYDROSAP T14 | *** |
| MIAMI MEDIUM:SUSPENSION T14 vs. MIAMI MEDIUM:HYDROSAP T14 | ** |
| MIAMI MEDIUM:SUSPENSION T14 vs. MIAMI MEDIUM:HYDROSAP T28 | ** |
| MIAMI MEDIUM:SUSPENSION T14 vs. GF MEDIUM:HYDROSAP T14 | *** |
| MIAMI MEDIUM:SUSPENSION T14 vs. GF MEDIUM:HYDROSAP T28 | *** |
| MIAMI MEDIUM:SUSPENSION T28 vs. GF MEDIUM:HYDROSAP T14 | *** |
| MIAMI MEDIUM:HYDROSAP T14 vs. GF MEDIUM:HYDROSAP T14 | * |
| MIAMI MEDIUM:HYDROSAP T28 vs. GF MEDIUM:HYDROSAP T14 | * |
| GF MEDIUM:SUSPENSION T1 vs. GF MEDIUM:HYDROSAP T14 | *** |
| GF MEDIUM:SUSPENSION T14 vs. GF MEDIUM:HYDROSAP T14 | *** |
| GF MEDIUM:SUSPENSION T14 vs. GF MEDIUM:HYDROSAP T28 | * |
| GF MEDIUM:SUSPENSION T28 vs. GF MEDIUM:HYDROSAP T14 | *** |
| GF MEDIUM:SUSPENSION T28 vs. GF MEDIUM:HYDROSAP T28 | ** |

| **FIBROBLAST** | **P value** |
| --- | --- |
| MIAMI MEDIUM:SUSPENSION T1 vs. MIAMI MEDIUM:HYDROSAP T28 | * |
| MIAMI MEDIUM:SUSPENSION T1 vs. GF MEDIUM:SUSPENSION T1 | ** |
| MIAMI MEDIUM:SUSPENSION T1 vs. GF MEDIUM:HYDROSAP T14 | *** |
| MIAMI MEDIUM:SUSPENSION T14 vs. MIAMI MEDIUM:HYDROSAP T14 | *** |
| MIAMI MEDIUM:SUSPENSION T14 vs. GF MEDIUM:SUSPENSION T1 | *** |
| MIAMI MEDIUM:SUSPENSION T14 vs. GF MEDIUM:HYDROSAP T14 | *** |
| MIAMI MEDIUM:SUSPENSION T28 vs. MIAMI MEDIUM:HYDROSAP T14 | *** |
| MIAMI MEDIUM:SUSPENSION T28 vs. GF MEDIUM:SUSPENSION T1 | *** |
| MIAMI MEDIUM:SUSPENSION T28 vs. GF MEDIUM:HYDROSAP T14 | *** |
| MIAMI MEDIUM:HYDROSAP T14 vs. MIAMI MEDIUM:HYDROSAP T28 | *** |
| MIAMI MEDIUM:HYDROSAP T14 vs. GF MEDIUM:SUSPENSION T14 | * |
| MIAMI MEDIUM:HYDROSAP T14 vs. GF MEDIUM:SUSPENSION T28 | ** |
| MIAMI MEDIUM:HYDROSAP T14 vs. GF MEDIUM:HYDROSAP T28 | *** |
| MIAMI MEDIUM:HYDROSAP T28 vs. GF MEDIUM:SUSPENSION T1 | *** |
| MIAMI MEDIUM:HYDROSAP T28 vs. GF MEDIUM:SUSPENSION T14 | * |
| MIAMI MEDIUM:HYDROSAP T28 vs. GF MEDIUM:HYDROSAP T14 | *** |
| GF MEDIUM:SUSPENSION T1 vs. GF MEDIUM:hPIs in SUSPENSION T14 | *** |
| GF MEDIUM:SUSPENSION T1 vs. GF MEDIUM:SUSPENSION T28 | *** |
| GF MEDIUM:SUSPENSION T1 vs. GF MEDIUM:HYDROSAP T28 | *** |
| GF MEDIUM:SUSPENSION T14 vs. GF MEDIUM:HYDROSAP T14 | *** |
| GF MEDIUM:SUSPENSION T28 vs. GF MEDIUM:HYDROSAP T14 | *** |
| GF MEDIUM:HYDROSAP T14 vs. GF MEDIUM:HYDROSAP T28 | *** |

| **LAMININ** | **P value** |
| --- | --- |
| MIAMI MEDIUM:SUSPENSION T1 vs. GF MEDIUM:SUSPENSION T28 | * |
| MIAMI MEDIUM:SUSPENSION T28 vs. MIAMI MEDIUM:HYDROSAP T14 | * |
| MIAMI MEDIUM:SUSPENSION T28 vs. GF MEDIUM:HYDROSAP T14 | ** |
| MIAMI MEDIUM:HYDROSAP T14 vs. GF MEDIUM:SUSPENSION T28 | *** |
| MIAMI MEDIUM:HYDROSAP T14 vs. GF MEDIUM:HYDROSAP T28 | ** |
| MIAMI MEDIUM:HYDROSAP T28 vs. GF MEDIUM:HYDROSAP T14 | * |
| GF MEDIUM:SUSPENSION T1 vs. GF MEDIUM:HYDROSAP T14 | * |
| GF MEDIUM:SUSPENSION T14 vs. GF MEDIUM:HYDROSAP T14 | * |
| GF MEDIUM:SUSPENSION T28 vs. GF MEDIUM:HYDROSAP T14 | *** |
| GF MEDIUM:HYDROSAP T14 vs. GF MEDIUM:HYDROSAP T28 | ** |

| **COLLAGEN IV** | **P value** |
| --- | --- |
| MIAMI MEDIUM:SUSPENSION T1 vs. GF MEDIUM:HYDROSAP T14 | ** |
| MIAMI MEDIUM:SUSPENSION T14 vs. MIAMI MEDIUM:HYDROSAP T14 | ** |
| MIAMI MEDIUM:SUSPENSION T14 vs. GF MEDIUM:HYDROSAP T14 | *** |
| MIAMI MEDIUM:SUSPENSION T28 vs. MIAMI MEDIUM:HYDROSAP T14 | * |
| MIAMI MEDIUM:SUSPENSION T28 vs. GF MEDIUM:HYDROSAP T14 | ** |
| MIAMI MEDIUM:HYDROSAP T14 vs. GF MEDIUM:SUSPENSION T28 | *** |
| MIAMI MEDIUM:HYDROSAP T14 vs. GF MEDIUM:HYDROSAP T28 | ** |
| MIAMI MEDIUM:HYDROSAP T28 vs. GF MEDIUM:HYDROSAP T14 | * |
| GF MEDIUM:SUSPENSION T28 vs. GF MEDIUM:HYDROSAP T14 | *** |
| GF MEDIUM:HYDROSAP T14 vs. GF MEDIUM:HYDROSAP T28 | *** |

| **COLLAGEN I** | **P value** |
| --- | --- |
| MIAMI MEDIUM:SUSPENSION T1 vs. MIAMI MEDIUM:HYDROSAP T14 | * |
| MIAMI MEDIUM:SUSPENSION T1 vs. GF MEDIUM:HYDROSAP T14 | * |
| MIAMI MEDIUM:SUSPENSION T28 vs. GF MEDIUM:HYDROSAP T14 | * |
| MIAMI MEDIUM:HYDROSAP T14 vs. GF MEDIUM:SUSPENSION T28 | * |
| GF MEDIUM:SUSPENSION T28 vs. GF MEDIUM:HYDROSAP T14 | ** |

**p < 0.05; **p < 0.01; ***p < 0.001.*
